# Supplementary material for: Trait–fitness associations do not predict within-species phenotypic evolution over 2 million years
Source: Proc Biol Sci. 2021 Jan 20;288(1943):20202047. doi: 10.1098/rspb.2020.2047 (PMC7893266; doi:10.1098/rspb.2020.2047)
Supplement: Electronic supplementary material: supplementary figures and tables [file rspb20202047supp1.docx]

**Electronic supplementary material**

Emanuela Di Martino & Lee Hsiang Liow

**"Trait-fitness associations do not predict within-species phenotypic evolution over 2 million years"**

*Proceedings of the Royal Society B,* DOI: 10.1098/rspb.2020.0049

**Figure S1. Stereoscope photograph of *Antarctothoa tongima*.**


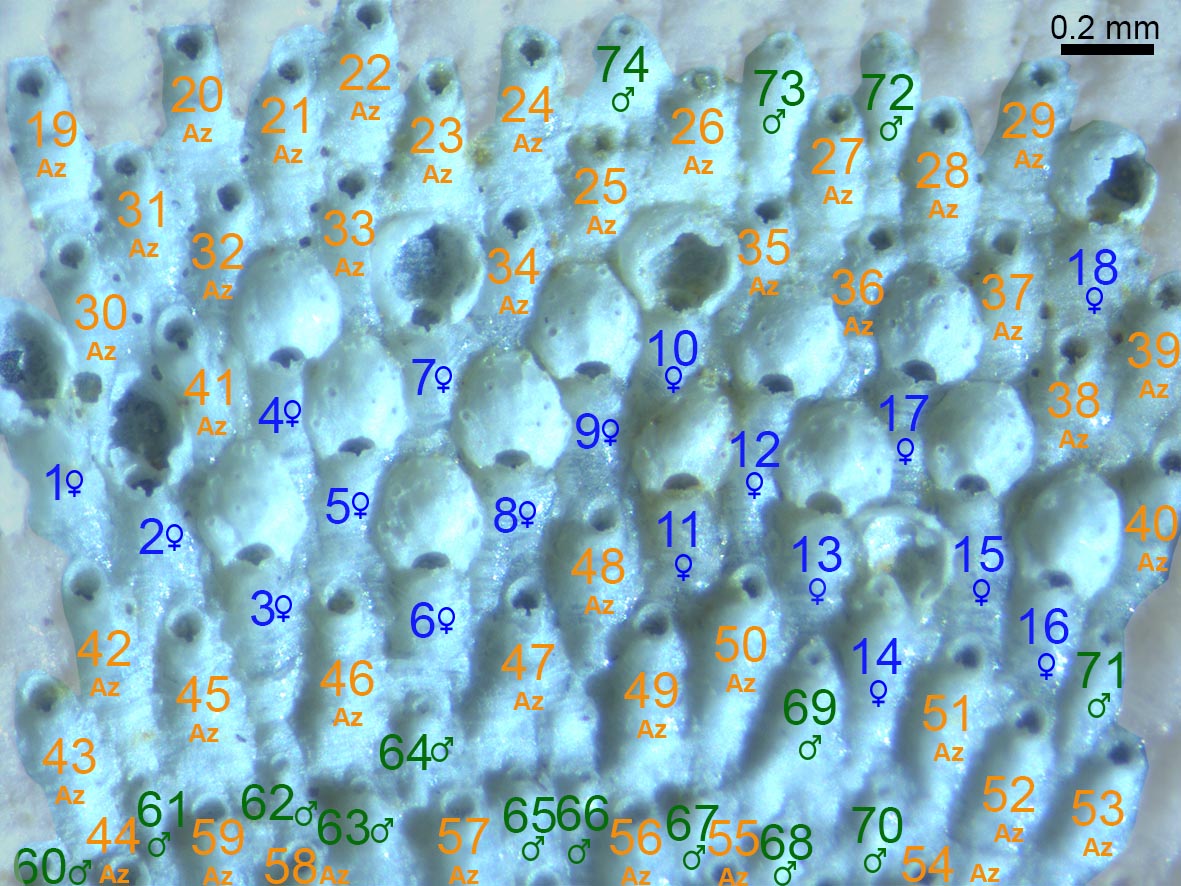
This is an example of a spot (*c.* 5 mm^2^) from which data are extracted. Numbered polymorphs were counted sequentially where Az are autozooids (orange), and related symbols indicate males (green) and females (blue). Grey shaded areas were removed to estimate areas effectively occupied by observed zooids. This specimen from the Nukumaru Limestone Formation is housed in the fossil collection (sample NKLS 89 Shell 12) of the Bryozoan Lab for Ecology, Evolution and Development (BLEED) at the Natural History Museum of Oslo (photograph number NKLS_89_#12I_Col_2_a). In our colonies we observed some males as intramural buds within either autozooids or female polymorphs, i.e. with a double opening (orifice) rim, with the male polymorph inside an autozooid orifice or a female polymorph orifice, but counted these as males, the last function they undertook.

**Figure S2. Competitive interactions among encrusting bryozoan colonies.**


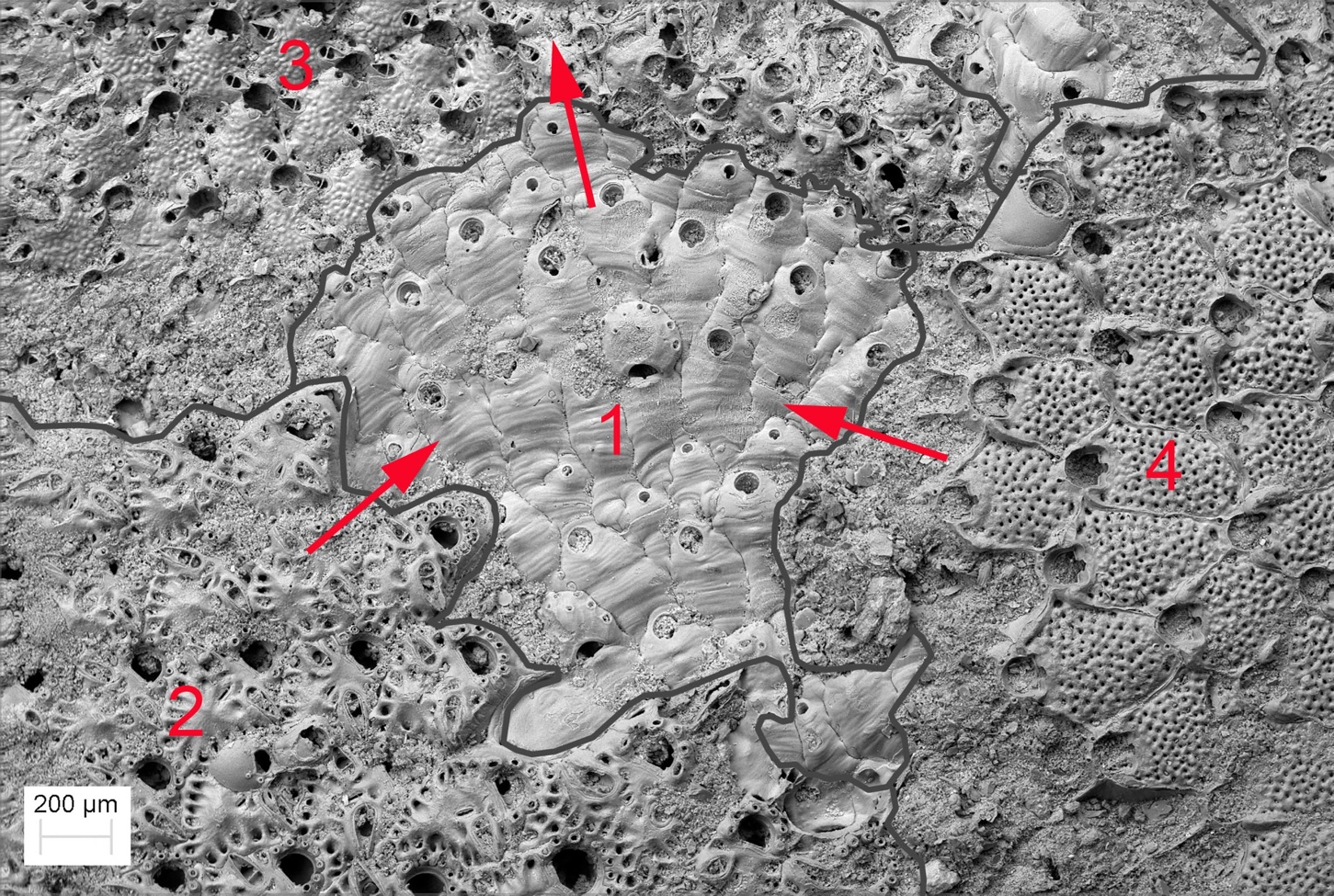
A scanning electron micrograph of part of an encrusted substrate from the Upper Kai-iwi Shell Bed. (1) *Antarctothoa tongima* is overgrown by (2) *Calloporina angustipora* and (4) *Schizosmittina* sp., and overgrows (3) *Exochella armata*. (2) also overgrows (3). This unregistered specimen is housed in the bryozoan palaeontological collection of the Natural History Museum London (NHMUK) (SEM micrograph number pdt16453).

**Table S1. Number of replicates with fecundity estimates**. Spots refer to a digital image of a part of a Colony (e.g. figure S1), one or several spots make up a Colony. Shells are substrates on which one or more colonies may have lived, and Samples refer to location samples from which shells are available. Numbers reported are the number of replicates from each formation. Note that the replicates come from time-averaged samples (i.e. the samples could have some from any time within the range of the age of the formation reported in millions of years (Ma) in the formation column). Isotope stage numbers and approximate ages of formations from Naish et al. (1998).

| Formation | Spot | Colony | Shell | Sample |
| --- | --- | --- | --- | --- |
| Nukumaru Limestone 2.29–2.08 Ma | 125 | 97 | 57 | 23 |
| Nukumaru Brown Sand 2.03–1.97 Ma | 93 | 58 | 38 | 15 |
| Lower Kai-iwi Shellbed 0.92–0.90 Ma | 84 | 73 | 47 | 15 |
| Upper Kai-iwi Shellbed 0.68–0.62 Ma | 67 | 45 | 35 | 9 |
| Shakespeare Cliff Basal Sand Shellbed 0.43–0.40 Ma | 125 | 88 | 32 | 6 |
| Landguard Formation 0.33–0.30 Ma | 65 | 53 | 31 | 8 |
| Total | 559 | 414 | 240 | 70 |

**Table S2. Number of replicates with measurements.**

Table format as in table S1.

| Formation | Spot | Colony | Shell | Sample |
| --- | --- | --- | --- | --- |
| Nukumaru Limestone 2.29–2.08 Ma | 94 | 72 | 41 | 22 |
| Nukumaru Brown Sand 2.03–1.97 Ma | 64 | 43 | 28 | 13 |
| Lower Kai-iwi Shellbed 0.92–0.90 Ma | 54 | 47 | 43 | 9 |
| Upper Kai-iwi Shellbed 0.68–0.62 Ma | 43 | 32 | 27 | 8 |
| Shakespeare Cliff Basal Sand Shellbed 0.43–0.40 Ma | 107 | 78 | 31 | 6 |
| Landguard Formation 0.33–0.30 Ma | 46 | 38 | 25 | 7 |
| Total | 408 | 311 | 186 | 65 |

**Figure S3. Measures of ovicell area are highly correlated.** Two different estimates of ovicell area (μm^2^) are highly correlated, even though different in absolute values. The one on the y-axis is presented in the analyses.

**
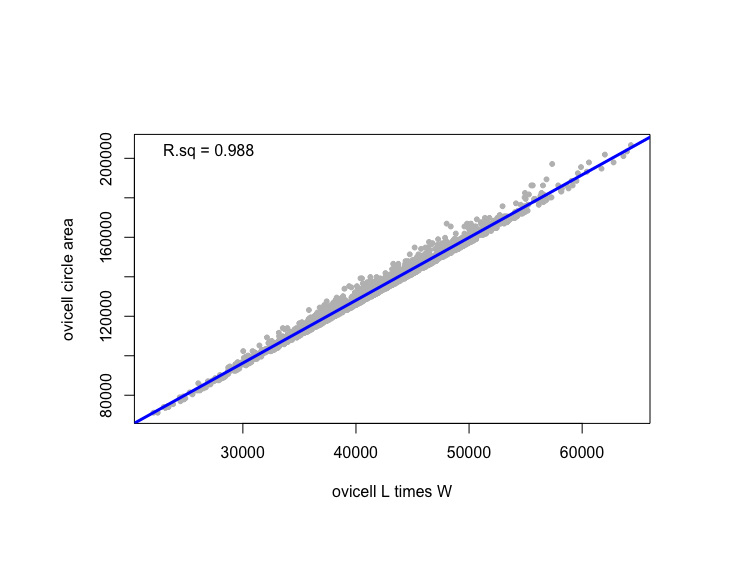
**

**Figure S4. Repeatability of size measurements.** Repeated measurements of areas are plotted in μm^2^ for both ovicells (top row) and autozooids (bottom row). The R-squares are all at least 0.97, indicating high repeatability.

**
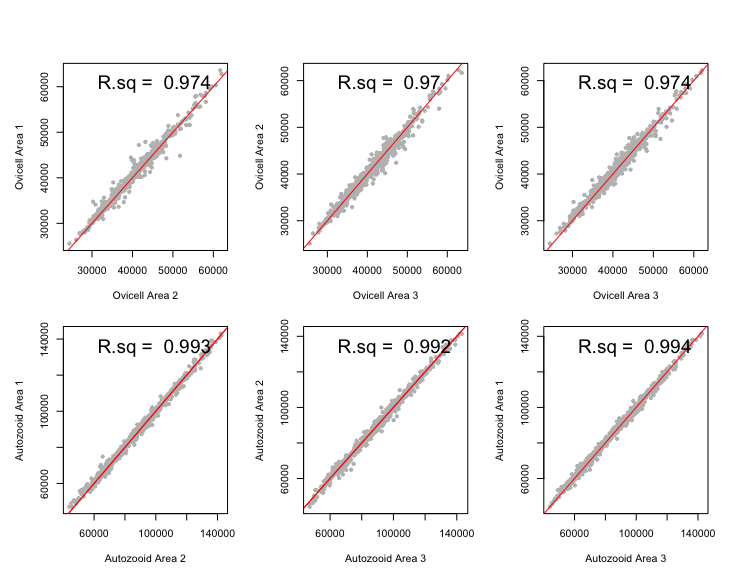
**

**Figure S5. Autozooid length, width and area.** Autozooid length (μm) and width (μm) correlate more highly with area (μm^2^) than with each other. Autozooid area correlates more with width than with length.

**
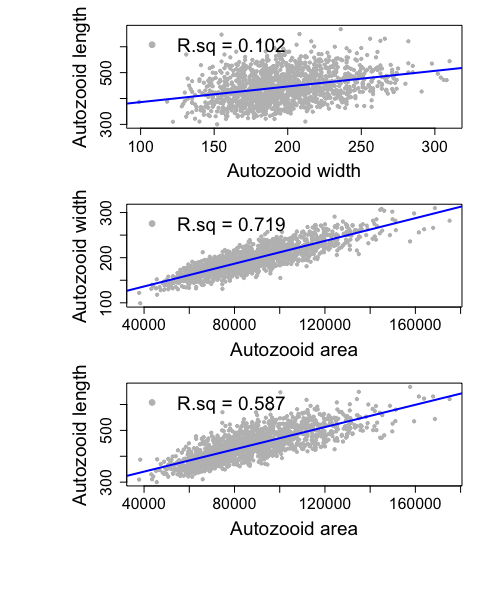
**

**Figure S6. Compare variances of traits within and across colonies.**

For each trait, we plot a histogram to show the distribution of trait variances within colonies. The solid vertical black line shows the median (of colony variances), the red vertical line the variance for all the data, and black arrows show variances within formations (see figures S7 to S9 for details). For both autozooid area and ovicell area, the variance across samples is greater that the median of colonies, although there are a few colonies that are very variable (right side of histogram).


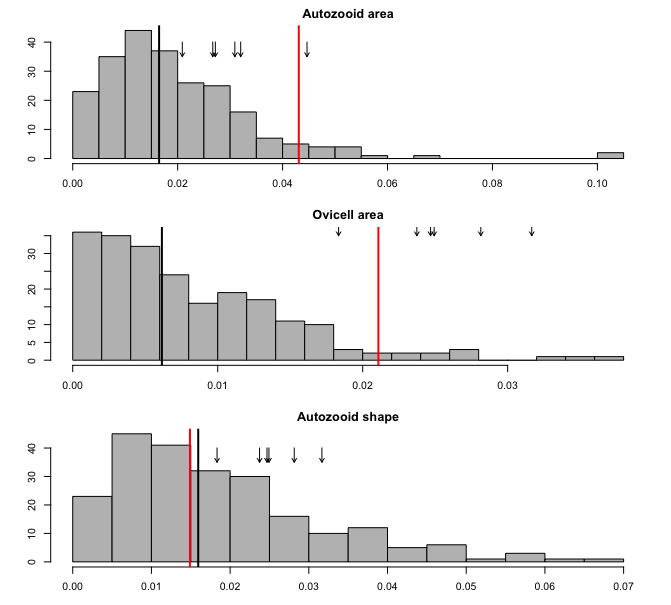


**Figure S7. Compare variances of autozooid area within and across colonies in each formation.** Histograms show the distribution of autozooid area variances within colonies. The solid vertical black line shows the median (of colony variances) in the formation, the blue vertical line the variance for all the data within the formation.


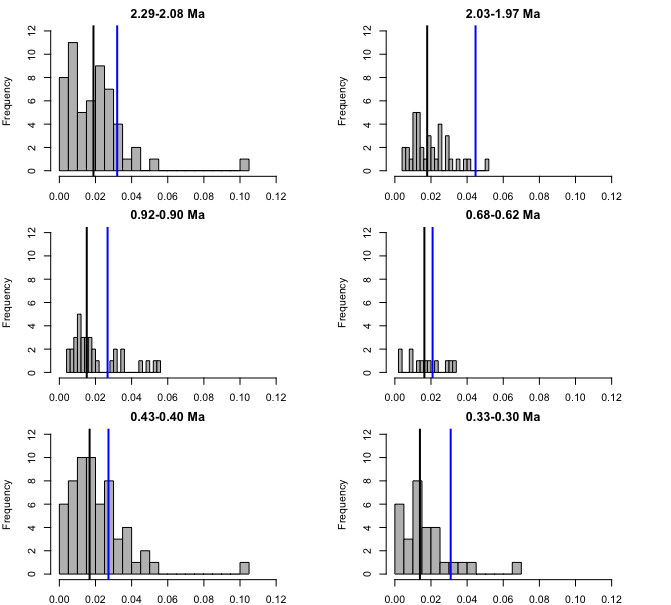


**Figure S8. Compare variances of ovicell areas within and across colonies in each formation.** Histograms show the distribution of ovicell area variances within colonies. The solid vertical black line shows the median (of colony variances) in the formation, the blue vertical line the variance for all the data within the formation.


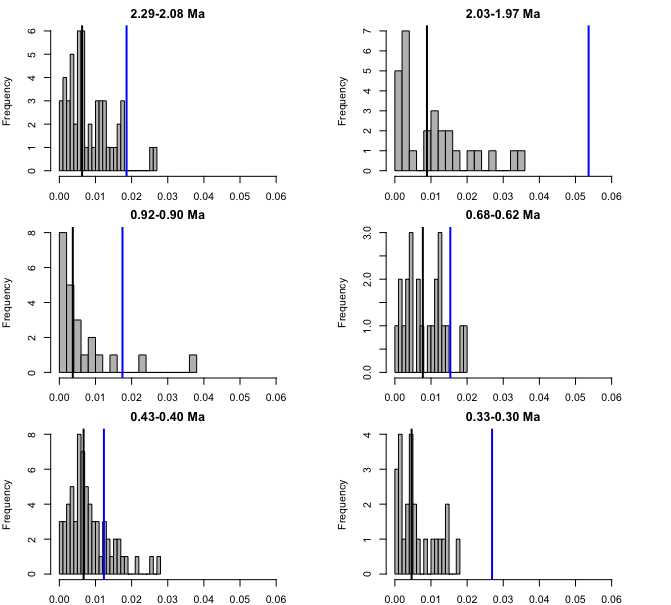


**Figure S9. Compare variances of autozooid shape within and across colonies in each formation.** Histograms show the distribution of autozooid shape variances within colonies. The solid vertical black line shows the median (of colony variances) in the formation, the blue vertical line the variance for all the data within the formation.


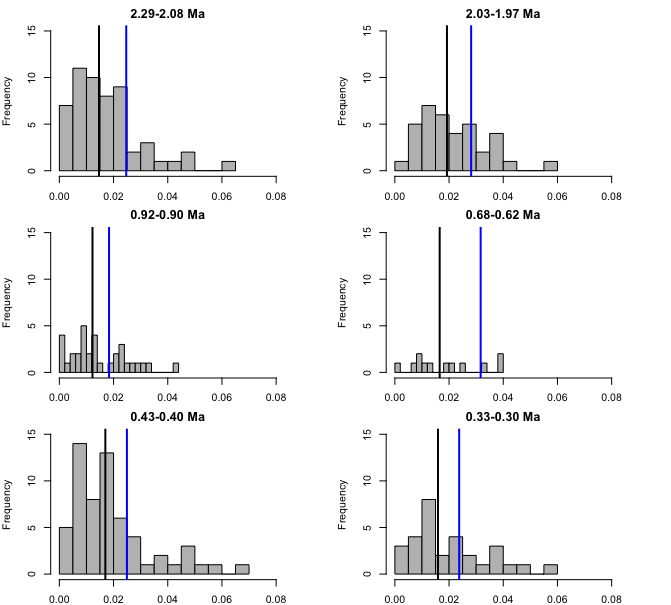


**Figure S10. Relationship between time interval length and variability of traits.** Each panel shows, for the three different traits, the relationship between the duration of the time interval and the standard deviation of the traits among the colonies from that time interval. There is no detected correlation for any of the traits (Autozooid area Spearman’s rho = 0.44, p = 0.38; Ovicell area Spearman’s rho = 0.26, p = 0.621; Autozooid shape Spearman’s rho = 0.59, p = 0.22). Note that the longest time interval (Nukumaru Limestone, duration 0.21 million years, see red circles) does not have a particularly high standard deviation for any of the traits.

**Table S3. Effect of time-averaging on variances.**

We compared the trait variances of samples (each row represents a unique sample) with ten or more colonies to the variances of other samples in the same formations using an f-test, Fligner test and Barlett test, each with slightly different assumptions. The p-values of each test are presented for the sample and trait. These samples span a much short period of time than the formation as a whole as they are collected at a specific location with precise GPS coordinates. Although there is no detailed age model for each of these samples, the time represented can range from 0.0006 to 0.06 million years. These coarse estimates are based on the thickness and duration of each formation (Rust 2008, PhD thesis), assuming the sedimentation rate in the basin was constant and that each samples was collected from a spot extending 1 m in thickness. However, shellbeds (from which our samples were collected) can also reflect a single, but more often multiple, relatively brief, events of concentration (Kidwell & Brenchley 1996).

| **Formation (Trait)** | **f-test** | **Fligner** | **Barlett** |
| --- | --- | --- | --- |
| Lower Kai-iwi Shellbed (Autozooid area) | 0.199 | 0.202 | 0.180 |
| Shakespeare Cliff Basal Sand Shellbed (Autozooid area) | 0.295 | 0.406 | 0.326 |
| Shakespeare Cliff Basal Sand Shellbed (Autozooid area) | 0.867 | 0.768 | 0.823 |
| Nukumaru Brown Sand (Autozooid area) | 0.689 | 0.714 | 0.679 |
| Landguard Formation (Autozooid area) | 0.183 | 0.357 | 0.174 |
| Shakespeare Cliff Basal Sand Shellbed (Ovicell area) | 0.820 | 0.900 | 0.840 |
| Shakespeare Cliff Basal Sand Shellbed (Ovicell area) | 0.741 | 0.817 | 0.820 |
| Nukumaru Brown Sand (Ovicell area) | 0.113 | 0.227 | 0.113 |
| Shakespeare Cliff Basal Sand Shellbed (Ovicell area) | 0.912 | 0.342 | 0.995 |
| Landguard Formation (Ovicell area) | 0.093 | 0.557 | 0.101 |
| Lower Kai-iwi Shellbed (Autozooid shape) | 0.019 | 0.023 | 0.017 |
| Shakespeare Cliff Basal Sand Shellbed (Autozooid shape) | 0.198 | 0.162 | 0.178 |
| Shakespeare Cliff Basal Sand Shellbed (Autozooid shape) | 0.392 | 0.415 | 0.425 |
| Nukumaru Brown Sand (Autozooid shape) | 0.296 | 0.274 | 0.289 |
| Landguard Formation (Autozooid shape) | 0.598 | 0.395 | 0.581 |

**References for table S3**

Kidwell, S.M. & Brenchley, P.J. (1996) Evolution of the fossil record: thickness trends in marine skeletal accumulations and their implications. In Jablonski, D., Erwin, D.H. & Lipps, J.H. (eds.) Evolutionary Paleobiology. University of Chicago Press, pp. 290­–336.

Rust, S. (2008) Plio-Pleistocene bryozoan faunas of the Wanganui Basin, New Zealand. Diversity, distribution and paleoecology. PhD thesis, University of Auckland, New Zealand.

**Table S4. Multivariate binomial glm trait-fitness model comparison.**

AIC-ranked binomial models of gravid females (ovicells) per polymorph (fecundity) as a function of three multivariate mean standardized traits (natural logged). z1 = log autozooid area (measured in μm^2^); z2 = log ovicell area (measured in μm^2^); z3 = log autozooid shape (dimensionless). The models for relative fitness are estimated as fecundity and are indicated in the model column, followed by their weight, ΔAICc, AICc and their log likelihood value.

| model | weight | Δ AICc | AICc | logLik |
| --- | --- | --- | --- | --- |
| z1+z2+z3+z1^2^ | 0.350 | 0.000 | 1315.051 | -652.342 |
| z1+z2+z3+ z1^2^+ z3^2^ | 0.210 | 1.018 | 1316.070 | -651.776 |
| z1+z2+z3+ z1^2^+ z2^2^ | 0.197 | 1.146 | 1316.197 | -651.839 |
| z1+z2+z3+ z1^2^+z2^2^+z3^2^ | 0.115 | 2.221 | 1317.272 | -651.288 |
| z1+z2+z3 | 0.047 | 4.003 | 1319.054 | -655.405 |
| z1+z2+z3+z2^2^ | 0.040 | 4.317 | 1319.369 | -654.500 |
| z1+z2+z3+z3^2^ | 0.022 | 5.563 | 1320.615 | -655.123 |
| z1+z2+z3+z2^2^+z3^2^ | 0.018 | 5.893 | 1320.945 | -654.213 |

**Table S5. Best multivariate binomial glm trait-fitness model.**

Model estimates of the best trait-fitness model from table S4. This model is plotted in main text figure 3.

|  | Estimate | Standard Error | z value | p-value |
| --- | --- | --- | --- | --- |
| Intercept | -180.580 | 74.718 | -2.417 | 0.016 |
| z1 | 358.205 | 149.963 | 2.389 | 0.017 |
| z2 | 2.359 | 2.282 | 1.034 | 0.301 |
| z3 | 1.045 | 0.223 | 4.682 | 0.000 |
| z1^2^ | -182.619 | 75.278 | -2.426 | 0.015 |

**Figure S11. Univariate trait-fitness binomial glm (ovicell area).**

The average number of gravid females (ovicells) per polymorph are plotted against average log autozooid areas (μm^2^) for each colony (grey dots, N = 244). Time intervals are indicated in Ma (= millions of years ago) on top of each panel. Solid black and grey lines show the prediction, and their 95% CI, respectively. Numbers within each panel are estimates with their standard errors in parentheses. Two estimates are significantly negative at a p < 0.05 level (indicated by *). Four time intervals show positive slopes while two show negative ones.


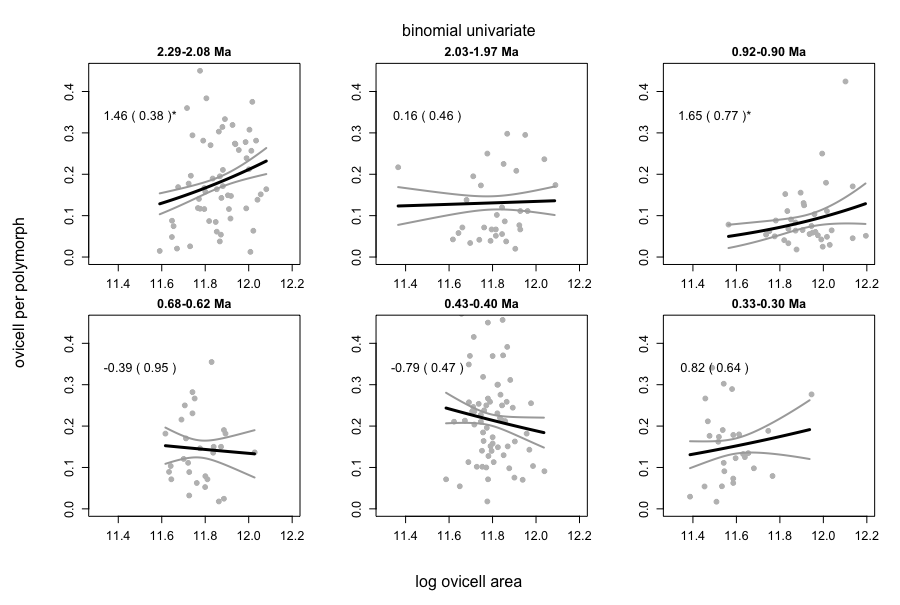


**Figure S12. Best Poisson multivariate trait-fitness model.**

The panels show fitted Poisson (glm) multivariate trait-fitness relationships from the best AIC-ranked model (table S6), combining data from all time intervals (N = 169). Relative fitness (fecundity) is approximated with the average number of gravid females (ovicells) per unit area, and the values on the x axes are natural logged and mean standardized. **A**, Plots autozooid area (originally measured in μm^2^); **B**, ovicell areas (originally measured in μm^2^); and **C**, autozooid shape, which is dimensionless. Grey dots are colony level data, black lines are predicted values and grey lines the 95% CI. Parameter estimates from this model are given in table S7.


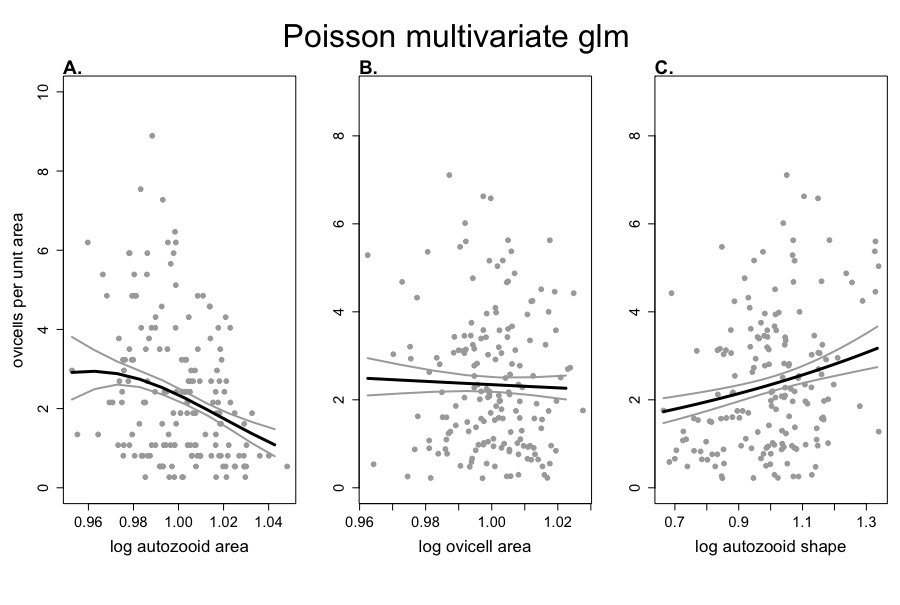


**Figure S13. Univariate trait-fitness Poisson glm (autozooid area).**

The average number of gravid females (ovicells) per unit area are plotted against standardized average log autozooid areas (μm^2^) for each colony (grey dots, N = 230). Time intervals are indicated in Ma (= millions of years ago) on top of each panel. Solid black and grey lines show the predictions and their 95% CI, respectively. Numbers within each panel are estimates with their standard errors in parentheses. Five estimates are significantly negative at a p < 0.05 level (indicated by *).


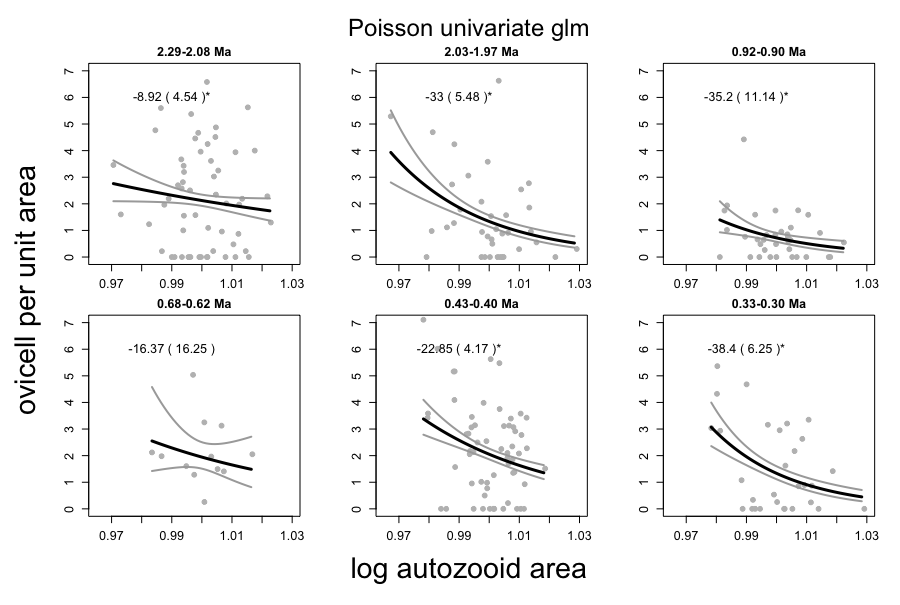


**Figure S14. Univariate trait-fitness Poisson glm (ovicell area).**

The average number of gravid females (ovicells) per unit area are plotted against standardized average log ovicell areas (μm^2^) for each colony (grey dots, N = 244). Time intervals are indicated in Ma (= millions of years ago) on top of each panel. Solid black and grey lines show the predictions and their 95% CI, respectively. Numbers within each panel are estimates with their standard errors in parentheses. Two estimates (each with a different sign) are significant at a p < 0.05 level (indicated by *). There are three positive and three negative estimates.


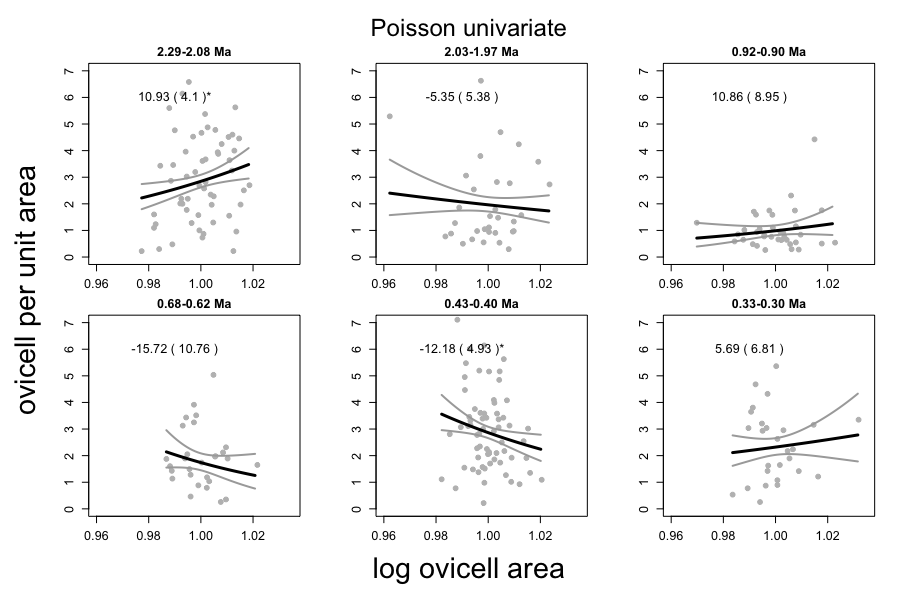


**Figure S15. Univariate trait-fitness Poisson glm (autozooid shape).**

The average number of gravid females (ovicells) per unit area are plotted against standardized average log autozooid shape for each colony (grey dots, N = 230). Time intervals are indicated in Ma (= millions of years ago) on top of each panel. Solid black and grey lines show the predictions and their 95% CI, respectively. Numbers within each panel are estimates with their standard errors in parentheses. Five estimates are significantly positive at a p < 0.05 level (indicated by *).


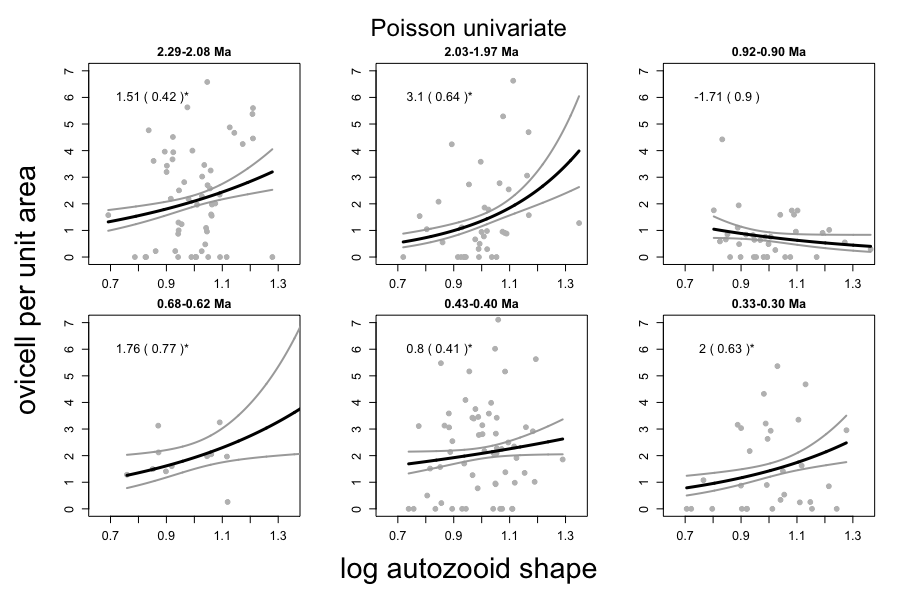


**Figure S16. Best linear (OLS) multivariate trait-fitness model.**

The panels show linear multivariate trait-fitness relationships from the best AIC-ranked model (table S8), combining data from all time intervals (N = 169). Relative fitness (fecundity) is approximated with the standardized number of gravid females (ovicells) per unit area, and the values on the x axes are natural logged and mean standardized. **A**, Plots autozooid area (originally measured in m^2^); **B**, ovicell areas (originally measured in m^2^); and **C**, autozooid shape, which is dimensionless. Grey dots are average colony level data, black lines are predicted slopes and grey lines the 95% CI. Parameter estimates from this model are given in table S9.
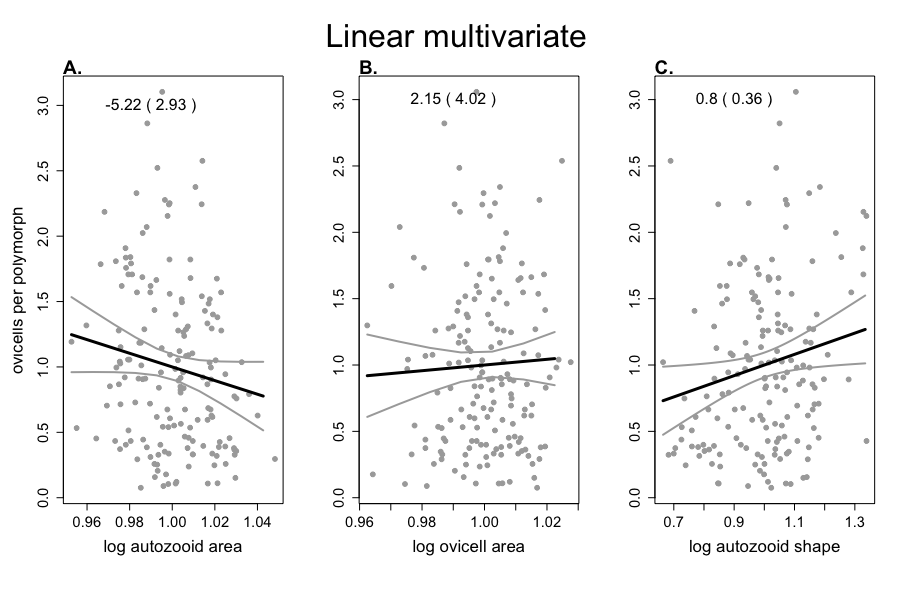


**Figure S17. Univariate trait-fitness linear (OLS) model (autozooid area).**

The standardized number of gravid females (ovicells) per unit area are plotted against standardized average log autozooid areas (μm^2^) for each colony (grey dots, N = 230). Time intervals are indicated in Ma (= millions of years ago) on top of each panel. Solid black and grey lines show the predicted slopes and their 95% CI, respectively. Numbers within each panel are estimated slopes with their standard errors in parentheses. Five slopes are negative and two of these are significantly so at a p < 0.05 level (indicated by *).


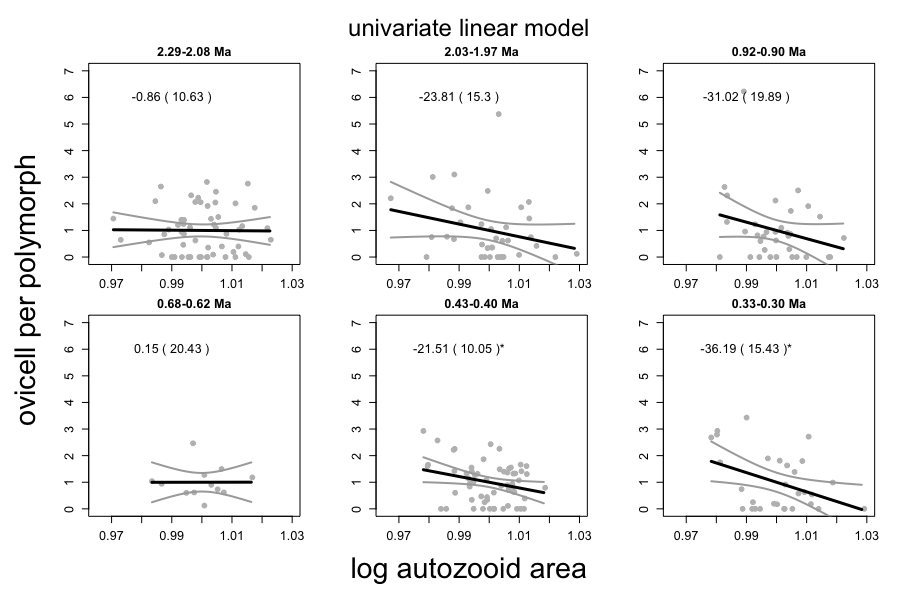


**Figure S18. Univariate trait-fitness linear (OLS) model (ovicell area).**

The standardized number of gravid females (ovicells) per unit area are plotted against standardized average log ovicell areas (μm^2^) for each colony (grey dots, N = 244). Time intervals are indicated in Ma (= millions of years ago) on top of each panel. Solid black and grey lines show the predicted slopes and their 95% CI, respectively. Numbers within each panel are estimated slopes with their standard errors in parentheses. None of the slopes are significant at the p < 0.05 level.


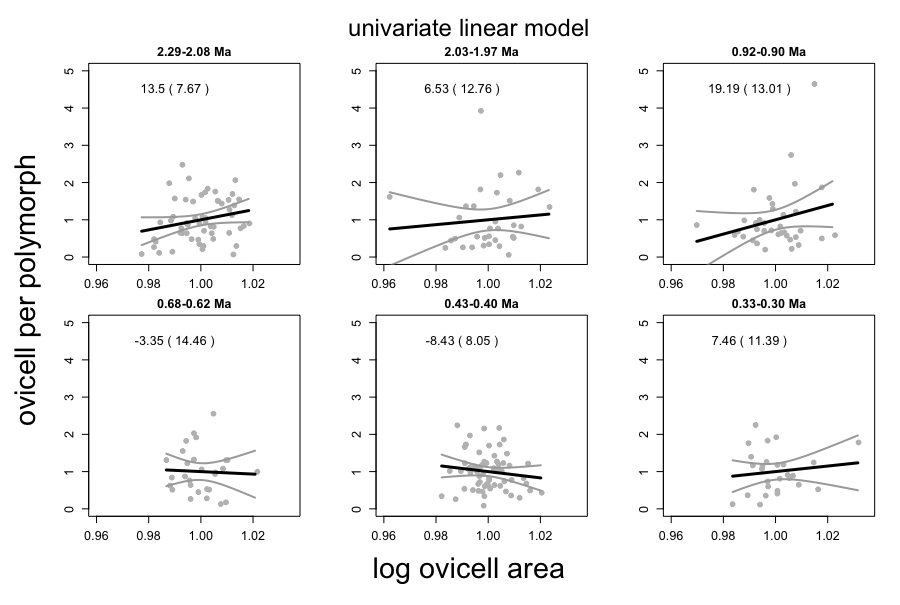


**Figure S19.** **Univariate trait-fitness linear (OLS) model (autozooid shape).**

The standardized number of gravid females (ovicells) per unit area are plotted against standardized average log autozooid shape for each colony (grey dots, N = 230). Time intervals are indicated in Ma (= millions of years ago) on top of each panel. Solid black and grey lines show the predicted slopes and their 95% CI, respectively. Numbers within each panel are estimated slopes with their standard errors in parentheses. All except one slope is positive although none are significant at the p < 0.05 level.


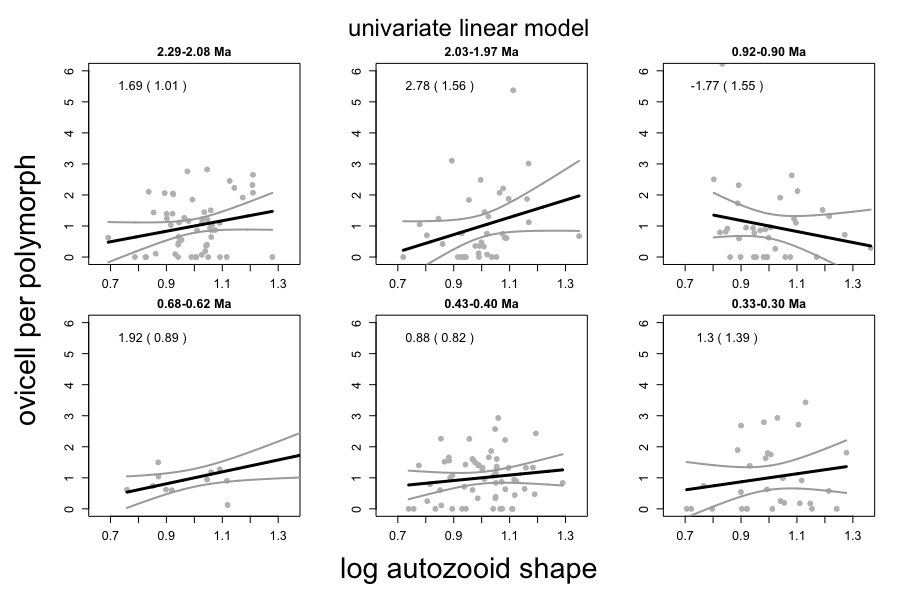


**Figure S20. Relationship between autozooid area and strength of trait-fitness association for binomial model.** There is no correlation between the size of model estimate (strength) and the mean log autozooid shape for each time interval (Pearson’s correlation = 0.6413 (-0.708, 0.881); Spearman’s rho = 0.257, p-value = 0.658).

**
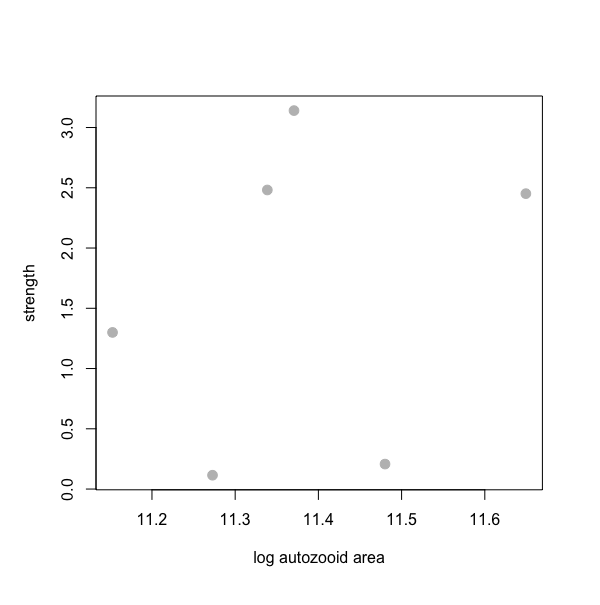
**

**Figure S21.** **Relationship between ovicell area and strength of trait-fitness association for binomial model.** There is no correlation between the size of model estimate (strength) and the mean log ovicell area for each time interval (Pearson’s correlation = 0.430 (-0.586, 0.920); Spearman’s rho = 0.486, p-value = 0.356).

**
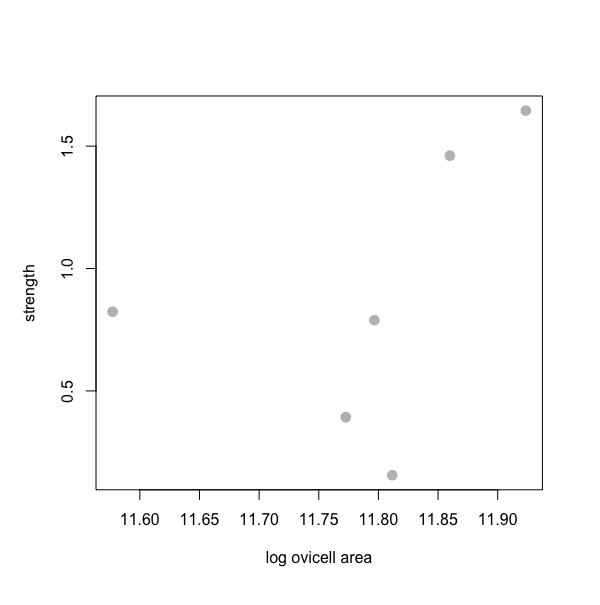
**

**Figure S22. Relationship between autozooid shape and strength of trait-fitness association for binomial model.** There is no correlation between the size of model estimate (strength) and the mean log autozooid shape for each time interval (Pearson’s correlation = -0.156 (-0.859, 0.751); Spearman’s rho = 0.2, p-value = 0.714)


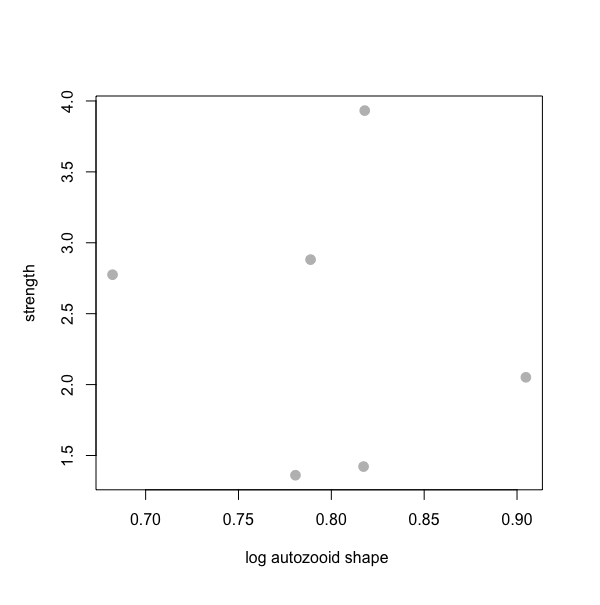


**Figure S23.** **Relationship between autozooid area and strength of trait-fitness association for Poisson model.** There is no correlation between the size of model estimate (strength) and the mean log autozooid shape for each time interval (Pearson’s correlation = 0.486 (-0.538, 0.930); Spearman’s rho = 0.371, p-value = 0.497)


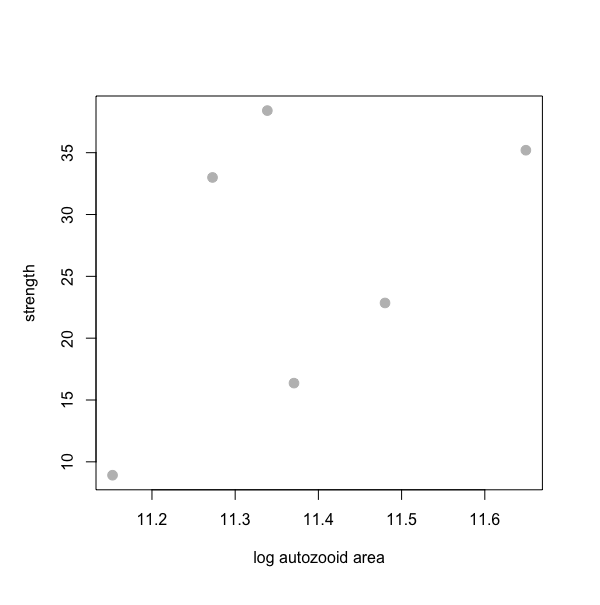


**Figure S24.** **Relationship between ovicell area and strength of trait-fitness association for Poisson model.** There is no correlation between the size of model estimate (strength) and the mean log ovicell area for each time interval (Pearson’s correlation = 0.390 (-0.616, 0.913); Spearman’s rho = -0.143, p-value = 0.8028).


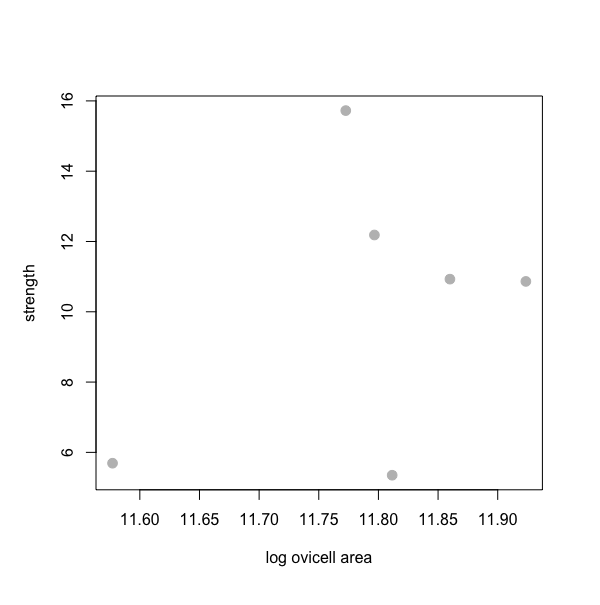


**Figure S25.** **Relationship between autozooid shape and strength of trait-fitness association for Poisson model.** There is no correlation between the size of model estimate (strength) and the mean log autozooid shape for each time interval (Pearson’s correlation = -0.063 (-0.832, 0.789); Spearman’s rho = -0.143, p-value = 0.802).

**
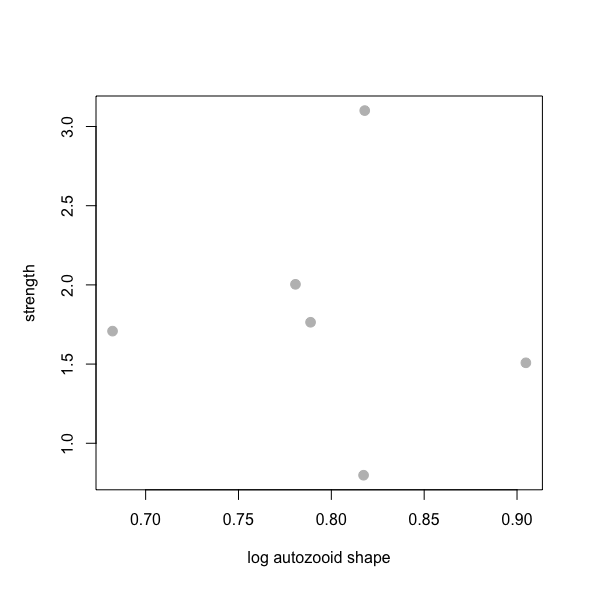
**

**Figure S26. Relationship between autozooid area and strength of trait-fitness association for the linear model.** There is no correlation between the linear model slope (“strength of selection”) and the mean log autozooid area for each time interval (Pearson’s correlation = 0.503 (-0.521, 0.933); Spearman’s rho = 0.2, p-value = 0.71).


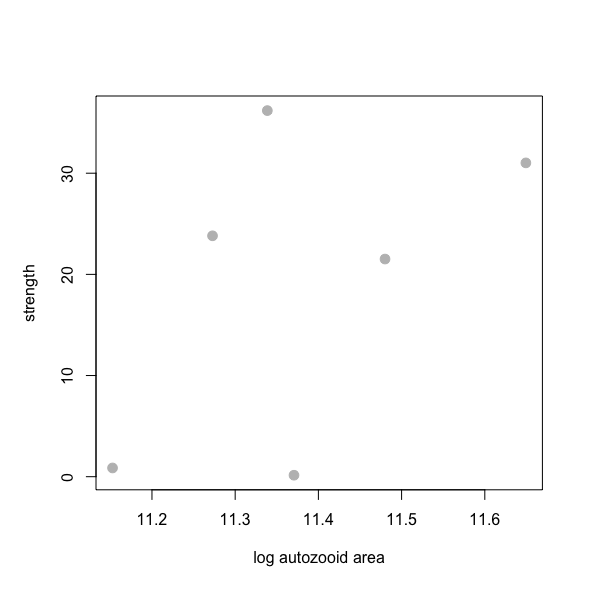


**Figure S27.** **Relationship between ovicell area and strength of trait-fitness association for the linear model.** There is no correlation between the linear model slope (“strength of selection”) and the mean log ovicell area for each time interval (Pearson’s correlation = 0.613 (-0.394, 0.951); Spearman’s rho = 0.7, p-value = 0.14).


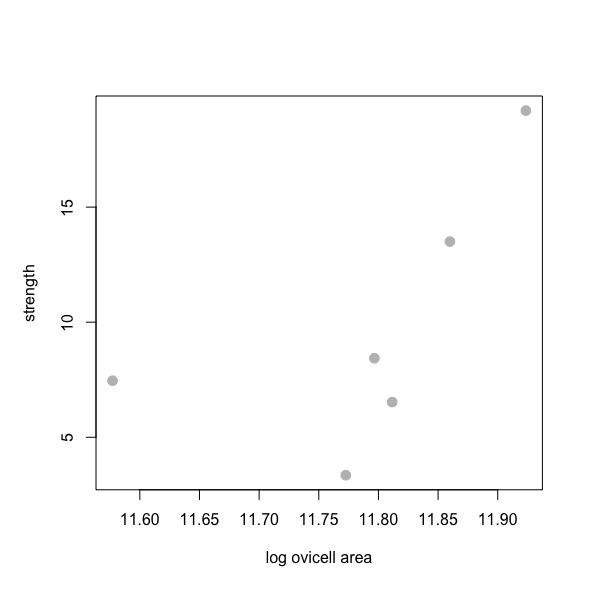


**Figure S28.** **Relationship between autozooid shape and strength of trait-fitness association for the linear model.** There is no correlation between the linear model slope (“strength of selection”) and the mean log autozooid shape for each time interval (Pearson’s correlation = 0.008 (-0.81 ,0.81); Spearman’s rho = 0.09, p-value = 0.91).

**
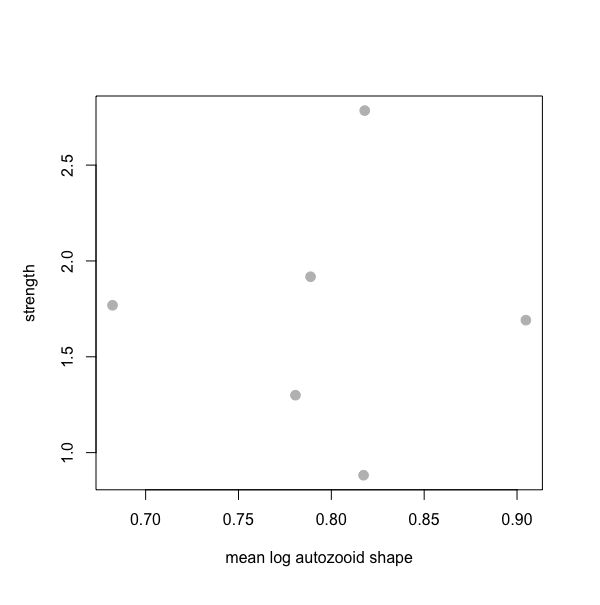
**

**Table S10. Model comparison for linear models of log autozooid area.**

We compared several additive linear models for log autozooid size (colony averages) using AIC model weights. Abbreviations are as follows: intersp.Interactions = number of interspecific interactions observed; intrasp.Interactions = number of intraspecific interactions observed; crowding = number of other bryozoan colonies observed on the same substrate (no overgrowth observed); ∂18O = average ∂^18^O values in the time interval of observation, ∂18O.sd standard deviation of ∂^18^O values in the time interval of observation; Az.Shape= log autozooid shape; Ovi.area = log ovicell area.

| model | weight | **Δ**AICc | AICc | logLik |
| --- | --- | --- | --- | --- |
| intrasp.Interactions + intersp.Interactions + crowding + ∂18O + ∂18O.sd + Az.Shape + Ovi.area | 0.529 | 0.000 | -110.873 | 65.003 |
| Az.Shape + Ovi.area + ∂18O + ∂18O.sd | 0.471 | 0.234 | -110.640 | 61.579 |
| Az.Shape + Ovi.area | 0.000 | 28.821 | -82.052 | 45.148 |
| ∂18O + ∂18O.sd | 0.000 | 37.780 | -73.093 | 40.669 |
| ∂18O + ∂18O.sd + intrasp.Interactions + intersp.Interactions + crowding | 0.000 | 39.026 | -71.848 | 43.272 |
| intrasp.Interactions + intersp.Interactions + crowding | 0.000 | 64.790 | -46.083 | 28.225 |

**Figure S29. Recorded ecological interactions through time.**

The number of overgrowth interactions (including both intra- and interspecific) are plotted in grey and the total number of bryozoan colonies on the same substrate are plotted in blue (with log base 2 scale on the right y-axis) with a jitter for visibility. Numbers below the second row show the age ranges (Ma) of Nukumaru Limestone, Nukumaru Brown Sand, Lower Kai-iwi Shellbed, Upper Kai-iwi Shellbed, Shakespeare Cliff Basal Sand Shellbed and Landguard Formation, respectively.
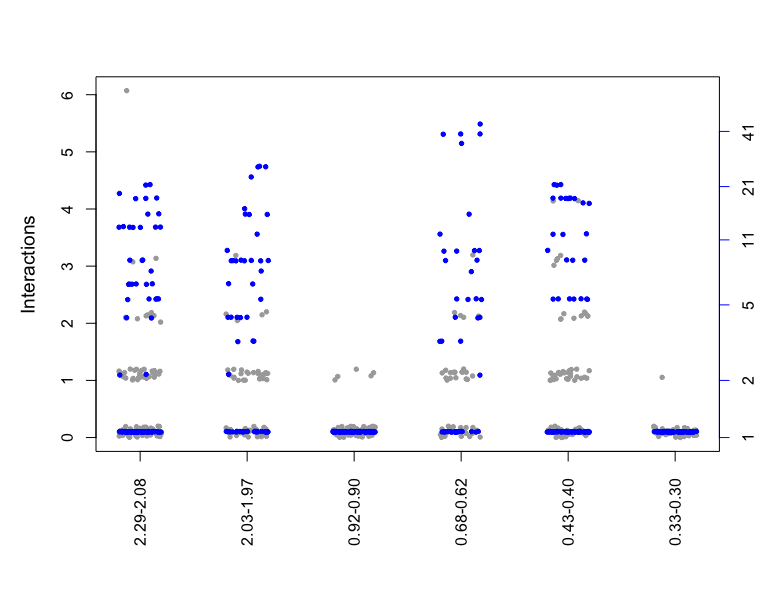


**Table S11. Model estimates for best model for log autozooid area.**

Estimates of the best model from table S10 are presented where explanatory variables significant at the p < 0.05 level are in bold. Note that we expect autozooid shape and ovicell area to have explanatory power on autozooid area, as indicated by their correlation (seen in the multivariate analyses above), hence they are included in these and the following models, but not further discussed in the main text, in the context of ecological interactions and palaeoenvironmental conditions.

|  | Estimate | Standard error | t value | p-value |
| --- | --- | --- | --- | --- |
| **Intercept** | 6.381 | 1.160 | 5.499 | 0.0000 |
| intrasp.Interactions | 0.008 | 0.021 | 0.386 | 0.6999 |
| intersp.Interactions | 0.021 | 0.038 | 0.560 | 0.5763 |
| crowding | -0.006 | 0.003 | -2.400 | 0.0175 |
| **∂18O** | 0.213 | 0.049 | 4.348 | 0.0000 |
| **∂18O.sd** | 0.674 | 0.116 | 5.796 | 0.0000 |
| **Az.Shape** | -0.653 | 0.119 | -5.473 | 0.0000 |
| **Ovi.area** | 0.385 | 0.095 | 4.055 | 0.0001 |

**Table S12. Model comparison for linear models of log ovicell area.**

We compared several additive linear models for log ovicell size (colony averages) using AIC model weights. Abbreviations are as in table S10 with the addition of Az.area = log autozooid area.

| model | weight | **Δ**AICc | AICc | logLik |
| --- | --- | --- | --- | --- |
| intrasp.Interactions + intersp.Interactions + crowding + ∂18O + ∂18O.sd + Az.area + Az.Shape | 0.686 | 0.000 | -190.089 | 104.611 |
| Az.area + Az.Shape + ∂18O + ∂18O.sd | 0.311 | 1.582 | -188.507 | 100.513 |
| ∂18O + ∂18O.sd + intrasp.Interactions + intersp.Interactions + crowding | 0.002 | 12.053 | -178.036 | 96.366 |
| Az.area + Az.Shape | 0.001 | 13.504 | -176.586 | 92.415 |
| ∂18O + ∂18O.sd | 0.000 | 16.529 | -173.560 | 90.902 |
| intrasp.Interactions + intersp.Interactions + crowding | 0.000 | 18.305 | -171.784 | 91.076 |

**Table S13. Model estimates for best model for log ovicell area.**

Estimates of the best model from table S12 are presented where explanatory variables significant at the p < 0.05 level are in bold.

|  | Estimate | Standard error | t value | p-value |
| --- | --- | --- | --- | --- |
| Intercept | 9.309 | 0.680 | 13.684 | 0.0000 |
| **intrasp.Interactions** | 0.045 | 0.016 | 2.811 | 0.0056 |
| intersp.Interactions | 0.005 | 0.030 | 0.156 | 0.8764 |
| crowding | -0.002 | 0.002 | -0.879 | 0.3806 |
| ∂18O | -0.066 | 0.041 | -1.626 | 0.1059 |
| **∂18O.sd** | -0.404 | 0.096 | -4.213 | 0.0000 |
| **Az.area** | 0.241 | 0.059 | 4.055 | 0.0001 |
| Az.Shape | 0.137 | 0.102 | 1.338 | 0.1829 |

**Table S14.** **Model comparison for linear models of log autozooid shape.**

We compared several additive linear models for log autozooid shape (colony averages) using AIC model weights. Abbreviations are as in tables S10 and S12.

| model | weight | **Δ**AICc | AICc | logLik |
| --- | --- | --- | --- | --- |
| intrasp.Interactions + intersp.Interactions + crowding + ∂18O + ∂18O.sd + Az.area + Ovi.area | 0.949 | 0.000 | -279.618 | 149.375 |
| Az.area + Ovi.area + ∂18O + ∂18O.sd | 0.051 | 5.850 | -273.768 | 143.143 |
| Az.area + Ovi.area | 0.000 | 16.110 | -263.508 | 135.876 |
| ∂18O + ∂18O.sd + intrasp.Interactions + intersp.Interactions + crowding | 0.000 | 24.459 | -255.160 | 134.928 |
| ∂18O + ∂18O.sd | 0.000 | 29.254 | -250.365 | 129.304 |
| intrasp.Interactions + intersp.Interactions + crowding | 0.000 | 33.320 | -246.298 | 128.333 |

**Table S15.** **Model estimates for best model for log autozooid shape.**

Estimates of the best model from table S14 are presented where explanatory variables significant at the p < 0.05 level are in bold.

|  | Estimate | Standard Error | t value | p-value |
| --- | --- | --- | --- | --- |
| **Intercept** | 2.710 | 0.737 | 3.675 | 0.0003 |
| **intrasp.Interactions** | 0.038 | 0.012 | 3.097 | 0.0023 |
| intersp.Interactions | 0.018 | 0.023 | 0.777 | 0.4384 |
| **crowding** | -0.004 | 0.002 | -2.483 | 0.0141 |
| ∂18O | -0.038 | 0.031 | -1.218 | 0.2251 |
| ∂18O.sd | 0.102 | 0.077 | 1.317 | 0.1897 |
| **Az.area** | -0.240 | 0.044 | -5.473 | 0.0000 |
| Ovi.area | 0.080 | 0.060 | 1.338 | 0.1829 |

**Table S16.** **Model comparison for fecundity.**

We compared several additive binomial glm models for fecundity (gravid female zooids given the number of total polymorphs) using AIC model weights. Abbreviations are as in tables S10 and S12.

| model | weight | **Δ**AICc | AICc | logLik |
| --- | --- | --- | --- | --- |
| Az.area + Az.shape + Ovi.area + ∂18O + ∂18O.sd | 1.000 | 0.000 | 1203.057 | -595.269 |
| ∂18O + ∂18O.sd + intrasp.Interactions + intersp.Interactions + crowding | 0.000 | 63.089 | 1266.146 | -626.814 |
| ∂18O + ∂18O.sd | 0.000 | 100.367 | 1303.424 | -648.639 |
| intrasp.Interactions + intersp.Interactions + onShell | 0.000 | 110.347 | 1313.404 | -652.580 |
| Az.area + Az.shape + Ovi.area | 0.000 | 115.997 | 1319.054 | -655.405 |
| intrasp.Interactions + intersp.Interactions + crowding + ∂18O + ∂18O.sd + Az.area + Az.shape + Ovi.area | 0.000 | 12660.093 | 13863.150 | -6923.125 |

**Table S17. Model estimates for best model for fecundity.**

Estimates of the best model from table S16 are presented where explanatory variables significant at the p < 0.05 level are in bold.

|  | Estimate | Standard Error | t value | p-value |
| --- | --- | --- | --- | --- |
| Intercept | 2.761 | 2.738 | 1.008 | 0.313 |
| **Az.area** | -1.492 | 0.188 | -7.952 | 0.000 |
| **Az.shape** | 0.854 | 0.279 | 3.062 | 0.002 |
| **Ovi.area** | 0.800 | 0.209 | 3.829 | 0.000 |
| **∂18O** | 0.397 | 0.119 | 3.322 | 0.001 |
| **∂18O.sd** | 2.979 | 0.294 | 10.121 | 0.000 |
