# Supplementary material for: Trait–fitness associations do not predict within-species phenotypic evolution over 2 million years
Source: Proc Biol Sci. 2021 Jan 20;288(1943):20202047. doi: 10.1098/rspb.2020.2047 (PMC7893266; doi:10.1098/rspb.2020.2047)
Supplement: Supplementary material: Code and data [file rspb20202047supp2.docx]

---

title: "Supplementary Information for Antarctothoa tongima ms"

output:

html_document:

df_print: paged

word_document: default

pdf_document: default

---

Version 19.8.2020 by L.H.Liow, final updates 3.11.2020

Data required: Antarctothoa_dataset_07.08.2019.xlsx

and

O18.csv

**Fig. S1. Stereoscope photograph of Antarctothoa tongima**

["Supplemental_figure_NKLS_89_#12I_Col_2_a.psd"]

This is an example of a spot (ca. 5 mm2) from which data are extracted. Numbered zooids were counted sequentially where Az are autozooids (orange), and males (blue) and females (pink) are indicated in their symbols. Shadowed areas were removed to calculate autozooid, male and female densities.

**Fig. S2. Competitive interactions between encrusting bryozoan colonies**

["S_figure.jpg"]

A Scanning Electron Micrograph of part of an encrustd substrate from Upper Kai-iwi Shellbed. (1) Antarctoa tongima is overgrown by (2) Calloporina angustipora and (4) Schizosmittina sp., and overgrows (3) Exochella armata. (2) also overgrows (3).

```{r, echo=FALSE, warnings=FALSE}

rm(list=ls())

setwd("/Users/leehl/Documents/Emanuela di Martino/Antarctothoa/")

library(openxlsx)

library(plyr)

library(knitr)

library(lme4)

library(MuMIn)

library(boot)

#FUNCTIONS

make.pretty=function(x){

x.table<-as.data.frame(x)

x.table$Model<-rownames(x.table)

for(i in 1:nrow(x.table))

x.table$Model[i]<- as.character(formula(paste(x.table$Model[i])))[3]

subset(x.table, select=c("Model", "weight", "delta","AICc", "logLik" ))}

logit=function(x) log(x/(1-x))

invlogit=function(x) 1/(1+exp(-x))

# #FUNCTIONS

#Read counts

Data= read.xlsx("Antarctothoa_dataset_07.08.2019.xlsx", 1)

Data$Females=(Data$N_ovicells+Data$N_female_non_ovicellate)

Data$reproductive=(Data$Females+Data$N_males)

Data$frame=(Data$Area_mm2-Data$Empy_Area_mm2)

#Spot level info

Data$Den.Zoo=(Data$N_zooids/Data$frame) #All zooids per mm sq

Data$Den.AZ=(Data$N_autozooids/Data$frame) #Auto zooids per mm sq

Data$Den.MZ=(Data$N_males/Data$frame) # Male zooids per mm sq

Data$Den.FZ=(Data$Females/Data$frame)# Female zooids per mm sq

Data$Den.Repro=(Data$reproductive/Data$frame)# reproductive zooids per mm sq

Data$Unique.Colony=paste(Data$Sample_ID, Data$Shell_ID, Data$Colony_ID)

Data$Unique.Colony=as.factor(Data$Unique.Colony)

Data$Unique.Shell=paste(Data$Sample_ID, Data$Shell_ID)

Data$Unique.Shell=as.factor(Data$Unique.Shell)

Data$Fec1=Data$N_ovicells/Data$frame

Data$Fec2=Data$N_ovicells/Data$N_zooids

```

**Number of replicates with fecundity estimates in each formation**

````{r, echo=FALSE}

t1=as.data.frame(table(Data$Formation_name))

t2=as.data.frame(tapply(Data$Unique.Colony, Data$Formation_name ,function(x){length(unique(x))}) )

t3=as.data.frame(tapply(Data$Unique.Shell, Data$Formation_name ,function(x){length(unique(x))}) )

t4=as.data.frame(tapply(Data$Sample_ID, Data$Formation_name ,function(x){length(unique(x))}) )

table=cbind(cbind(t1,t2),cbind(t3,t4))

table=table[,2:5]

colnames(table)=c("Spot", "Colony", "Shell", "Sample")

rownames(table)=c("Landguard Formation", "Lower Kai-iwi Shellbed" ,"Nukumaru Brown Sand", "Nukumaru Limstone", "Shakespeare Cliff Basal Sand Shellbed", "Upper Kai-iwi Shellbed")

table=table[c(4,3,2,6,5,1),] #rearrange

table=rbind(table, colSums(table))

rownames(table)[7]=c("Total")

labels= c("2.29 to 2.08 ", "2.03 to 1.97","0.92 to 0.9", "0.68 to 0.62", "0.43 to 0.4", "0.33 to 0.30 ", "")

table=cbind(table, labels)

colnames(table)=c("Spot", "Colony", "Shell", "Sample", "Max to min age (Ma)")

kable(table)

```

```{r , echo=FALSE}

#Read measurements

Measurements= read.xlsx("Antarctothoa_dataset_07.08.2019.xlsx", 2)

Measurements$Unique.Colony=paste(Measurements$Sample_ID, Measurements$Shell_ID, Measurements$Colony_ID)

Measurements$Unique.Colony=as.factor(Measurements$Unique.Colony)

Measurements$Image_ID=as.factor(Measurements$Image_ID)

Measurements$Unique.Shell=paste(Measurements$Sample_ID, Measurements$Shell_ID)

Measurements$Unique.Shell=as.factor(Measurements$Unique.Shell)

Measurements$Zooid.area=(Measurements$Zooid_length*Measurements$Zooid_width)

Measurements$Ovicell.area=(Measurements$Ovicell_length*Measurements$Ovicell_width)

Measurements$Ovicell.circle=(pi*(0.5*(Measurements$Ovicell_length+Measurements$Ovicell_width))^2)

```

**Number of replicates with area measurements in each formation**

```{r, echo=FALSE}

t3=as.data.frame(tapply(Measurements$Unique.Shell, Measurements$Formation_name ,function(x){length(unique(x))}))

t2=as.data.frame(tapply(Measurements$Unique.Colony, Measurements$Formation_name ,function(x){length(unique(x))}) )

t1=as.data.frame(tapply(Measurements$Image_ID, Measurements$Formation_name ,function(x){length(unique(x))}) )

t4=as.data.frame(tapply(Measurements$Sample_ID, Measurements$Formation_name ,function(x){length(unique(x))}) )

table=cbind(cbind(t1,t2),cbind(t3,t4))

colnames(table)=c("Spot", "Colony", "Shell", "Sample")

table=rbind(table, colSums(table))

rownames(table)=c("Landguard Formation", "Lower Kai-iwi Shellbed" ,"Nukumaru Brown Sand", "Nukumaru Limstone", "Shakespeare Cliff Basal Sand Shellbed", "Upper Kai-iwi Shellbed", "Total")

table=table[c(4,3,2,6,5,1,7),]

kable(table)

```

```{r, echo=FALSE}

#Make Colony averages for both Measurements and Counts (Data)

#Join data from density and measurements. Data matrix is called "joined".

#females are either measured as auto zooids or ovicells but not both

Measurements$Zooid.shape=(Measurements$Zooid_length/Measurements$Zooid_width)

Area.Zooid=tapply(Measurements$Zooid.area,Measurements$Unique.Colony, median, na.rm=TRUE)

Area.Zooid=as.data.frame(Area.Zooid)

Area.Zooid$Unique.Colony=rownames(Area.Zooid)

Shape.Zooid=tapply(Measurements$Zooid.shape,Measurements$Unique.Colony, median, na.rm=TRUE)

Shape.Zooid=as.data.frame(Shape.Zooid)

Shape.Zooid$Unique.Colony=rownames(Shape.Zooid)

L.Zooid=tapply(Measurements$Zooid_length,Measurements$Unique.Colony, median, na.rm=TRUE)

L.Zooid=as.data.frame(L.Zooid)

L.Zooid$Unique.Colony=rownames(L.Zooid)

W.Zooid=tapply(Measurements$Zooid_width,Measurements$Unique.Colony, median, na.rm=TRUE)

W.Zooid=as.data.frame(W.Zooid)

W.Zooid$Unique.Colony=rownames(W.Zooid)

Area.Ovicell=tapply(Measurements$Ovicell.area,Measurements$Unique.Colony, median, na.rm=TRUE)

Area.Ovicell=as.data.frame(Area.Ovicell)

Area.Ovicell$Unique.Colony=rownames(Area.Ovicell)

Area.Ovicell.c=tapply(Measurements$Ovicell.circle,Measurements$Unique.Colony, median, na.rm=TRUE)

Area.Ovicell.c=as.data.frame(Area.Ovicell.c)

Area.Ovicell.c$Unique.Colony=rownames(Area.Ovicell.c)

joined0=join(Area.Zooid,Area.Ovicell.c, by="Unique.Colony", type="full")

joined1=join(L.Zooid, W.Zooid, by="Unique.Colony", type="full")

joined2=join (joined1,joined0, by="Unique.Colony", type="right")

joined2=join (joined2,Shape.Zooid, by="Unique.Colony", type="right")

Colony.Sample_ID=tapply(Data$Sample_ID,Data$Unique.Colony, function(x){paste(x[1])})

Colony.Sample_ID=as.data.frame(Colony.Sample_ID)

Colony.Sample_ID$Unique.Colony=rownames(Colony.Sample_ID)

Colony.reproductive=tapply(Data$reproductive,Data$Unique.Colony, mean, na.rm=TRUE)

Colony.reproductive=as.data.frame(Colony.reproductive)

Colony.reproductive$Unique.Colony=rownames(Colony.reproductive)

Colony.N_zooids=tapply(Data$N_zooids,Data$Unique.Colony, mean, na.rm=TRUE)

Colony.N_zooids=as.data.frame(Colony.N_zooids)

Colony.N_zooids$Unique.Colony=rownames(Colony.N_zooids)

Colony.N_zooids$Colony.N_zooids=ceiling(Colony.N_zooids$Colony.N_zooids)#

Colony.N_autozooids=tapply(Data$N_autozooids,Data$Unique.Colony, mean, na.rm=TRUE)

Colony.N_autozooids=as.data.frame(Colony.N_autozooids)

Colony.N_autozooids$Unique.Colony=rownames(Colony.N_autozooids)

Colony.Den.AZ=tapply(Data$Den.AZ,Data$Unique.Colony, mean, na.rm=TRUE)

Colony.Den.AZ=as.data.frame(Colony.Den.AZ)

Colony.Den.AZ$Unique.Colony=rownames(Colony.Den.AZ)

Colony.Den.Repro=tapply(Data$Den.Repro,Data$Unique.Colony, mean, na.rm=TRUE)

Colony.Den.Repro=as.data.frame(Colony.Den.Repro)

Colony.Den.Repro$Unique.Colony=rownames(Colony.Den.Repro)

Colony.Fec1=tapply(Data$Fec1,Data$Unique.Colony, mean, na.rm=TRUE)

Colony.Fec1=as.data.frame(Colony.Fec1)

Colony.Fec1$Unique.Colony=rownames(Colony.Fec1)

Colony.Fec2=tapply(Data$Fec2,Data$Unique.Colony, mean, na.rm=TRUE)

Colony.Fec2=as.data.frame(Colony.Fec2)

Colony.Fec2$Unique.Colony=rownames(Colony.Fec2)

Colony.Ovi=tapply(Data$N_ovicells,Data$Unique.Colony, mean, na.rm=TRUE)

Colony.Ovi=as.data.frame(Colony.Ovi)

Colony.Ovi$Unique.Colony=rownames(Colony.Ovi)

Colony.Ovi$Ovi=ceiling(Colony.Ovi$Colony.Ovi)# make integer

Colony.Form=tapply(Data$Formation_name,Data$Unique.Colony, function(x){paste(x[1])})

Colony.Form=as.data.frame(Colony.Form)

Colony.Form$Unique.Colony=rownames(Colony.Form)

Colony.Shell=tapply(Data$Unique.Shell,Data$Unique.Colony, function(x){paste(x[1])})

Colony.Shell=as.data.frame(Colony.Shell)

Colony.Shell$Unique.Colony=rownames(Colony.Shell)

Colony.area=tapply(Data$frame,Data$Unique.Colony, mean, na.rm=TRUE)

Colony.area=as.data.frame(Colony.area)

Colony.area$Unique.Colony=rownames(Colony.area)

Colony=join(Colony.Fec1,Colony.Fec2, by="Unique.Colony", type="full")

Colony=join(Colony, Colony.reproductive, by="Unique.Colony", type="right")

Colony=join(Colony,Colony.N_zooids, by="Unique.Colony", type="right")

Colony=join(Colony,Colony.N_autozooids, by="Unique.Colony", type="right")

Colony=join(Colony,Colony.Den.AZ, by="Unique.Colony", type="right")

Colony=join(Colony,Colony.Den.Repro, by="Unique.Colony", type="right")

Colony=join(Colony,Colony.Form, by="Unique.Colony", type="right")

Colony=join(Colony,Colony.Shell, by="Unique.Colony", type="right")

Colony=join(Colony,Colony.Ovi, by="Unique.Colony", type="right")

Colony=join(Colony,Colony.area, by="Unique.Colony", type="right")

Colony=join(Colony,Colony.Sample_ID, by="Unique.Colony", type="right")

joined=join(joined2,Colony, by="Unique.Colony", type="right")

joined$Formation_name=as.factor(joined$Colony.Form)

Formation=levels(joined$Formation_name)

Formation=levels(joined$Formation_name)[c(4,3,2,6,5,1)]#in temporal order

time.labels=c("2.29-2.08 Ma", "2.03-1.97 Ma", "0.92-0.90 Ma", "0.68-0.62 Ma" , "0.43-0.40 Ma", "0.33-0.30 Ma")

```

```{r, echo=FALSE}

#Read interaction data

int=read.xlsx("Antarctothoa_dataset_07.08.2019.xlsx", 3)

int$Formation.order=0

for (i in 1:6){

int$Formation.order[which(int$Formation_name==Formation[i])]=i}

```

**Main Text Fig. 2**

**Temporal changes for A. tongima colonies over 2 million years **

The six time intervals are plotted with medians, the boxed interquartile ranges and the span of the data. A. Plots the number of gravid females (ovicells) per polymorph. B. Autozooid and C. ovicell area are both in natural log m2 and D. autozooid shape is dimensionless, but also natural logged. The data (colony averages or values) are grey points on the boxplots, with scatter for visibility. Numbers below the second row show the age ranges in million years of Nukumaru Limestone, Nukumaru Brown Sand, Lower Kai-iwi Shellbed, Upper Kai-iwi Shellbed, Shakespeare Cliff Basal Sand Shellbed and Landguard Formation, respectively.

```{r,echo=FALSE}

postscript(file="Tongima.Fig.2.eps", width=9, height=7, onefile = FALSE, horizontal =FALSE, paper = "special")

par(mfrow=c(2,2), oma=c(5,0,0,0))

par(mar=c(3,5,4,1))

joined$Formation.order=NA

for (i in 1:6){

joined$Formation.order[which(joined$Formation_name==Formation[i])]=i}

labels= c("2.29-2.08 ", "2.03-1.97 ","0.92-0.90 ", "0.68-0.62 ", "0.43-0.40 ", "0.33-0.30 ")

boxplot(joined$Colony.Fec2 ~ joined$Formation.order, xlab="" ,ylab="ovicells per polymorph",cex.lab=1.2,xaxt="n", range=0) #remember Fec2 is ovicells per unit zooid

points(joined$Colony.Fec2 ~ jitter(joined$Formation.order, factor=1), cex=0.5, pch=20, col="grey")

axis(1, labels = FALSE)

title("(a)", line = 0.5, adj=0, cex.main=1)

boxplot(log(joined$Area.Zooid) ~joined$Formation.order, xlab="",

ylab="log autozooid area",cex.lab=1.2,xaxt="n", range=0)

points(log(joined$Area.Zooid) ~ jitter(joined$Formation.order, factor=1), cex=0.5, pch=20, col="grey")

axis(1, labels = FALSE)

title("(b)", line = 0.5, adj=0, cex.main=1)

par(mar=c(5,5,2,1))

boxplot(log(joined$Area.Ovicell.c) ~ joined$Formation.order, xaxt="n", xlab="", ylab="log ovicell area",cex.lab=1.22, range=0)

points(log(joined$Area.Ovicell.c) ~ jitter(joined$Formation.order, factor=1), cex=0.5, pch=20, col="grey")

axis(1, labels = FALSE)

title("(c)", line = 0.5, adj=0, cex.main=1)

text(x = seq_along(labels), y = par("usr")[3]-0.0, srt = 0, adj = 1.2,srt=90,

labels = labels, xpd = TRUE, cex=1)

boxplot(log(joined$Shape.Zooid) ~ joined$Formation.order, xaxt="n", xlab="", ylab="log autozooid shape", cex.lab=1.2, range=0)

points(log(joined$Shape.Zooid) ~ jitter(joined$Formation.order, factor=1), cex=0.5, pch=20, col="grey")

axis(1, labels = FALSE)

title("(d)", line = 0.5, adj=0, cex.main=1)

text(x = seq_along(labels), y = par("usr")[3]-0.0, srt = 0, adj = 1.2,srt=90,

labels = labels, xpd = TRUE, cex=1)

dev.off()

```

**SI Fig. 21**

**Recorded ecological interactions through time. **

The number of overgrowth interactions (including both intra- and interspecific) are plotted in gray and the number of other bryozoan colonies on the same substrate are plotted in blue (with log base 2 scale on the right y-axis) with a jitter for visibility. Numbers below the second row show the age ranges (Ma) of Nukumaru Limestone, Nukumaru Brown Sand, Lower Kai-iwi Shellbed, Upper Kai-iwi Shellbed, Shakespeare Cliff Basal Sand Shellbed and Landguard Formation, respectively.

```{r,echo=FALSE}

par(mfrow=c(1,1), oma=c(0,0,0,0))

par(mar=c(8,5,4,3))

joined$Formation.order=NA

for (i in 1:6){

joined$Formation.order[which(joined$Formation_name==Formation[i])]=i}

plot(jitter(int$interactions+.1, factor=0.5) ~ jitter(int$Formation.order, factor=1), cex=0.9, pch=20, col="darkgrey", ylab="Interactions", xlab="", axes=F, cex.lab=1.2)

axis(2)

axis(1, tick=TRUE, labels=F)

text(x = seq_along(labels), y = par("usr")[3]-0.0, srt = 0, adj = 1.3,srt=90,

labels = labels, xpd = TRUE, cex=1)

#title("D.", line = 0.2, adj=0)

par(new=TRUE)

points(jitter(log2(int$onShell+1)+.1, factor=0.5) ~ jitter(int$Formation.order, factor=1), cex=0.9, pch=20, col="blue")

axis(4, at=c(log2(1), log2(2), log2(5),log2(11), log2(21), log2(41)),

labels=c(1,2, 5, 11,21, 41), col="blue")

box()

```

**Make a dataset without missing data**

The dataset has missing data. For multivariate analyses, we use a dataset with no missing data for any of the three traits. N=169. Note that z1 = log autozooid area, z2 = log ovicell area, z3 = log autozooid shape, a is colony area measured, t = zooids counted, f = number of ovicells counts and TIME random effects for time interval and colony respectively. ft=f/t

Note that z1 through three are averaged by the mean of the "population" so that the betas can be compared. ft is also standardized (relative to "population")

```{r, echo=FALSE}

no.na=na.omit(joined) #get rid of NA's

f.count=no.na$Ovi

t.count=no.na$Colony.N_zooids

a=no.na$Colony.area

TIME=as.factor(no.na$Formation_name)

f=no.na$Ovi/mean(no.na$Ovi)

t=no.na$Colony.N_zooids/mean(no.na$Colony.N_zooids)

ft=(no.na$Ovi/no.na$Colony.N_zooids)/mean((no.na$Ovi/no.na$Colony.N_zooids))

z1=log(no.na$Area.Zooid)/mean(log(no.na$Area.Zooid))

z2=log(no.na$Area.Ovicell.c)/mean(log(no.na$Area.Ovicell.c))

z3=log(no.na$Shape.Zooid)/mean(log(no.na$Shape.Zooid))

full=do.call(cbind, list(f,t,ft,z1,z2,z3,f.count, t.count, a,TIME))

colnames(full)=c("f","t","ft", "z1","z2","z3", "f.count", "t.count","a","TIME")

full=as.data.frame(full)

```

**Covariances of the three traits involved**

```{r, echo=FALSE}

z=cov2cor(cov(full[,3:6])) #covariances of fecundity and three traits

kable(z)

```

** Linear multivariate model (OLS) comparisons**

Best model is a simple lm(f/(t) ~ z1 + z2 + z3

SI Table S1:

AIC-ranked multivariate linear regression of colony-level relative fitness (female per poly morph, standardized by global mean) versus mean standardized traits (logged)

```{r, echo=FALSE}

res=lm(ft~z1+z2+z3, data=full)

res1=lm(ft~z1+z2+z3+I(z1^2)+I(z2^2)+I(z3^2), data=full)

res2=lm(ft~z1+z2+z3+I(z1^2), data=full)

res3=lm(ft~z1+z2+z3+I(z2^2), data=full)

res4=lm(ft~z1+z2+z3+I(z3^2), data=full)

res5=lm(ft~z1+z2+z3+I(z2^2)+I(z3^2), data=full)

res6=lm(ft~z1+z2+z3+I(z1^2)+I(z2^2), data=full)

res7=lm(ft~z1+z2+z3+I(z1^2)+I(z3^2), data=full)

compare=model.sel(res, res1, res2, res3, res4, res5, res6, res7)

kable(compare)

#make.pretty(compare)

#kable(make.pretty(compare))

#write.csv(file="res.csv",make.pretty(compare))

#write.csv(file="res.csv",summary(res)$coefficients)

```

**Time has info**

Compare best model with the same but with TIME random effetcs.

```{r, echo=FALSE}

res.TIME=lmer(ft~z1+z2+z3+(1|TIME), data=full)

AIC(res, res.TIME) #improved with time

```

**Estimates for best multivariate lm**

```{r, echo=FALSE}

kable(summary(res)$coefficients)

```

**Estimates for second multivariate lm**

```{r, echo=FALSE}

kable(summary(res2)$coefficients)

#write.csv(file="res.csv",summary(res)$coefficients)

```

** Linear multivariate model figure**

**SI Figure**

```{r, echo=FALSE}

par(mfrow=c(1,3), oma=c(3,1,3,0), mar=c(5,4,3,2))

plot(full$z1,full$ft,ylab="ovicells per polymorph", xlab="log autozooid area", main="", cex.axis=1.4, cex.lab=1.6, pch=20, col="darkgrey", cex=1.5)

z1.new=seq(min(full$z1),max(z1),0.01)

z2.new=rep(mean(full$z2), length(z1.new))

z3.new=rep(mean(full$z3), length(z1.new))

newdata=as.data.frame(list(z1=z1.new,z2=z2.new,z3=z3.new))

pred=predict(res,newdata=newdata,se.fit=TRUE)

lines(newdata$z1, (pred$fit), lwd=3)

lines(newdata$z1, (pred$fit-1.96*pred$se.fit),

col="darkgrey", lwd=2)

lines(newdata$z1, (pred$fit+1.96*pred$se.fit),

col="darkgrey", lwd=2)

text(0.99, 3, cex=1.5, paste(round(summary(res)$coefficients[2,1], digits=2), "(", round(summary(res)$coefficients[2,2], digits=2), ")"))

title("A.", line = 0.2, adj=0, cex.main=1.8)

plot(full$z2,full$ft, ylab="", xlab="log ovicell area",main="", cex.axis=1.4, cex.lab=1.6, pch=20, col="darkgrey", cex=1.5)

z2.new=seq(min(full$z2),max(z2),0.01)

z1.new=rep(mean(full$z1), length(z2.new))

z3.new=rep(mean(full$z3), length(z2.new))

newdata=as.data.frame(list(z1=z1.new,z2=z2.new,z3=z3.new))

pred=predict(res,newdata=newdata,se.fit=TRUE)

lines(newdata$z2, (pred$fit), lwd=3)

lines(newdata$z2, (pred$fit-1.96*pred$se.fit),

col="darkgrey", lwd=2)

lines(newdata$z2, (pred$fit+1.96*pred$se.fit),

col="darkgrey", lwd=2)

text(0.99, 3, cex=1.5,paste(round(summary(res)$coefficients[3,1], digits=2), "(", round(summary(res)$coefficients[3,2], digits=2), ")"))

title("B.", line = 0.2, adj=0, cex.main=1.8)

plot(z3,f/(t), ylab="", xlab="log autozooid shape", main="", cex.axis=1.4, cex.lab=1.6, pch=20, col="darkgrey", cex=1.5)

z3.new=seq(min(z3), max(z3), 0.01)

z2.new=rep(mean(z2),length(z3.new))

z1.new=rep(mean(z1), length(z3.new))

newdata=as.data.frame(list(z1=z1.new,z2=z2.new,z3=z3.new))

pred=predict(res,newdata=newdata,se.fit=TRUE)

lines(newdata$z3, (pred$fit), lwd=3)

lines(newdata$z3, (pred$fit-1.96*pred$se.fit),

col="darkgrey", lwd=2)

lines(newdata$z3, (pred$fit+1.96*pred$se.fit),

col="darkgrey", lwd=2)

text(0.9, 3, cex=1.5,paste(round(summary(res)$coefficients[4,1], digits=2), "(", round(summary(res)$coefficients[4,2], digits=2), ")"))

title("C.", line = 0.2, adj=0, cex.main=1.8)

mtext("Linear multivariate", side=3, outer=T, cex=2, padj=0.5)

```

**Diagnostics for LM**

Residuals cf fitted, more or less equal spread of residuals around horizontal. a couple of outliers (39, 121, 68)

Normal QQ plot some deviations at the ends. In general residuals are normal

Scale-location for residuals, ok indication of homoscedasticity.

Last plot "Residuals vs Leverage"" ok too

```{r, echo=FALSE}

par(mfrow=c(2,2))

plot(res)

```

**Univariate linear models: include all data now**

**z1**

```{r, echo=FALSE}

joined.no.na.a=joined[which(!is.na(joined$Area.Zooid)),] #remove NA's, N= 230

bet=NULL

z.mean=NULL

z.var=NULL

par(mfrow=c(2,3), oma=c(5,3,3,0), mar=c(2,4,2,2))

for (i in 1:length(Formation)){

temp=which(joined.no.na.a$Colony.Form==Formation[i])

temp=joined.no.na.a[temp,]

ft=temp$Colony.Fec2/mean(temp$Colony.Fec2)

z1=log(temp$Area.Zooid)/mean(log(temp$Area.Zooid))

res=lm(ft~z1, data=temp)

val=summary(res)$coefficients

val1=val[2,1]#slope

val2=val[2,2]#slope se

val[2,4]#slope p

sig=val[2,4]<0.05

#print(summary(res))

bet[i]=val1

z.mean[i]=mean(log(temp$Area.Zooid)) #original

z.var[i]=sd(log(temp$Area.Zooid))#original std

plot(z1,ft,ylab="", xlab="", main=paste(time.labels[i]), pch=20, col="grey", cex.axis=1.2, cex=1.5, xlim=c(0.965, 1.03), ylim=c(0,7))

text(11,0.5, labels[i], cex=1.3, col="darkgrey")

if (sig=="TRUE"){

text(0.99,6,cex=1.2,paste(round(val1 ,digits=2),"(", round(val2, digits=2), ")*"))

}else{

text(0.99,6,cex=1.2,paste(round(val1 ,digits=2),"(", round(val2, digits=2), ")"))

}

z1.new=seq(min(z1),max(z1),0.001)

newdata=as.data.frame(list(z1=z1.new))

pred=predict(res,newdata=newdata,se.fit=TRUE, type="response")

# Plot estimate:

lines(newdata$z1,(pred$fit), lwd=3)

lines(newdata$z1, (pred$fit-1.96*pred$se.fit), lwd=2, col="darkgrey")

lines(newdata$z1, (pred$fit+1.96*pred$se.fit), lwd=2, col="darkgrey")

}

mtext("log autozooid area" , side=1, outer=TRUE, cex=1.8, line=2)

mtext("ovicell per polymorph" , side=2, outer=TRUE, cex=1.8)

mtext("univariate linear model" , side=3, outer=TRUE, cex=1.5)

```

**No relationship between mean z1 and strength of selection**

```{r, echo=FALSE}

par(mfrow=c(1,1), mar=c(5,4,4,2), oma=c(1,1,1,1))

plot(z.mean , abs(bet) , col="grey", pch=20, cex=2, xlab="log autozooid area", ylab="strength")

x=lm(formula = abs(bet) ~ z.mean )

#abline(x$coefficients[1], x$coefficients[2])

summary(x)

cor.test(z.mean , abs(bet))

cor.test(z.mean , abs(bet), method="spearman")

```

**Univariate linear model z2: include all data now**

```{r, echo=FALSE}

joined.no.na.a=joined[which(!is.na(joined$Area.Ovicell.c)),] #remove NA's N = 244

bet=NULL

z.mean=NULL

z.var=NULL

par(mfrow=c(2,3), oma=c(5,3,3,0), mar=c(2,4,2,2))

for (i in 1:length(Formation)){

temp=which(joined.no.na.a$Colony.Form==Formation[i])

temp=joined.no.na.a[temp,]

ft=temp$Colony.Fec2/mean(temp$Colony.Fec2)

z2=log(temp$Area.Ovicell.c)/mean(log(temp$Area.Ovicell.c))

a=temp$Colony.area

res=lm(ft~z2, data=temp)

val=summary(res)$coefficients

val1=val[2,1]#slope

val2=val[2,2]#slope se

val[2,4]#slope p

sig=val[2,4]<0.05

#print(summary(res))

bet[i]=val1

z.mean[i]=mean(log(temp$Area.Ovicell.c)) #original

z.var[i]=sd(log(temp$Area.Ovicell.c))#original std

plot(z2,ft,ylab="", xlab="", main=paste(time.labels[i]), pch=20, col="grey", cex.axis=1.2, cex=1.5, ylim=c(0,5), xlim=c(0.96, 1.035))

text(11,0.5, labels[i], cex=1.3, col="darkgrey")

if (sig=="TRUE"){

text(0.99,4.5,cex=1.2,paste(round(val1 ,digits=2),"(", round(val2, digits=2), ")*"))

}else{

text(0.99,4.5,cex=1.2,paste(round(val1 ,digits=2),"(", round(val2, digits=2), ")"))

}

z2.new=seq(min(z2),max(z2),0.001)

a.new=rep(mean(a),length(z2.new))

newdata=as.data.frame(list(z2=z2.new, a=a.new))

pred=predict(res,newdata=newdata,se.fit=TRUE, type="response")

# Plot estimate:

lines(newdata$z2,(pred$fit), lwd=3)

lines(newdata$z2, (pred$fit-1.96*pred$se.fit), lwd=2, col="darkgrey")

lines(newdata$z2, (pred$fit+1.96*pred$se.fit), lwd=2, col="darkgrey")

}

mtext("log ovicell area" , side=1, outer=TRUE, cex=1.8, line=2)

mtext("ovicell per polymorph" , side=2, outer=TRUE, cex=1.8)

mtext("univariate linear model" , side=3, outer=TRUE, cex=1.5)

```

**No relationship between mean and strength of selection for z2**

```{r, echo=FALSE}

par(mfrow=c(1,1), mar=c(5,4,4,2), oma=c(1,1,1,1))

plot(z.mean , abs(bet) , col="grey", pch=20, cex=2, ylab="strength", xlab="log ovicell area")

x=lm(formula = abs(bet) ~ z.mean )

#abline(x$coefficients[1], x$coefficients[2])

summary(x)

cor.test(abs(bet) , z.mean)

cor.test(abs(bet) , z.mean, method="spearman")

```

**Univariate linear model: z3**

```{r, echo=FALSE}

joined.no.na.a=joined[which(!is.na(joined$Shape.Zooid)),] #remove NA's N = 230

bet=NULL

z.mean=NULL

z.var=NULL

par(mfrow=c(2,3), oma=c(5,3,3,0), mar=c(2,4,2,2))

for (i in 1:length(Formation)){

temp=which(joined.no.na.a$Colony.Form==Formation[i])

temp=joined.no.na.a[temp,]

ft=temp$Colony.Fec2/mean(temp$Colony.Fec2)

z3=log(temp$Shape.Zooid)/mean(log(temp$Shape.Zooid))

a=temp$Colony.area

res=lm(ft~z3, data=temp)

val=summary(res)$coefficients

val1=val[2,1]#slope

val2=val[2,2]#slope se

val[2,4]#slope p

sig=val[2,4]<0.05

#print(summary(res))

bet[i]=val1

z.mean[i]=mean(log(temp$Shape.Zooid)) #original

z.var[i]=sd(log(temp$Shape.Zooid))#original std

plot(z3,ft,ylab="", xlab="", main=paste(time.labels[i]), pch=20, col="grey", cex.axis=1.2, cex=1.5, xlim=c(0.65, 1.35), ylim=c(0,6))

text(11,0.5, labels[i], cex=1.3, col="darkgrey")

if (sig=="TRUE"){

text(0.85,5.5,cex=1.2,paste(round(val1 ,digits=2),"(", round(val2, digits=2), ")*"))

}else{

text(0.85,5.5,cex=1.2,paste(round(val1 ,digits=2),"(", round(val2, digits=2), ")"))

}

z3.new=seq(min(z3),max(z3),0.001)

a.new=rep(mean(a),length(z3.new))

newdata=as.data.frame(list(z3=z3.new, a=a.new))

pred=predict(res,newdata=newdata,se.fit=TRUE, type="response")

# Plot estimate:

lines(newdata$z3,(pred$fit), lwd=3)

lines(newdata$z3, (pred$fit-1.96*pred$se.fit), lwd=2, col="darkgrey")

lines(newdata$z3, (pred$fit+1.96*pred$se.fit), lwd=2, col="darkgrey")

}

mtext("log autozooid shape" , side=1, outer=TRUE, cex=1.8, line=2)

mtext("ovicell per polymorph" , side=2, outer=TRUE, cex=1.8)

mtext("univariate linear model" , side=3, outer=TRUE, cex=1.5)

```

**No relationship between mean and strength of selection for z3**

```{r, echo=FALSE}

par(mfrow=c(1,1), mar=c(5,4,4,2), oma=c(1,1,1,1))

plot(z.mean , abs(bet) , col="grey", pch=20, cex=2, ylab="strength", xlab="mean log autozooid shape")

x=lm(formula = abs(bet) ~ z.mean )

#abline(x$coefficients[1], x$coefficients[2])

summary(x)

cor.test(abs(bet) , z.mean)

cor.test(abs(bet) , z.mean, method="spearman")

```

**Compare sample variances for larger samples(>10) within formations with rest of given formation**

```{r, echo=FALSE}

temp=cbind.data.frame(joined$Area.Zooid, joined$Colony.Sample_ID, joined$Formation.order)

temp=na.omit(temp)

long=which(tapply(temp$`joined$Colony.Sample_ID`, temp$`joined$Colony.Sample_ID`, length)>10)

f.test=NA

barlett=NA

fligner=NA

f.name=NA

#now for each of those in "long", compare variance of the trait with the rest of the data

for (i in 1:length(long)){

f.name[i]=temp[which(temp$`joined$Colony.Sample_ID`==names(long)[i]),]$`joined$Formation.order`[1]

part=temp[which(temp$`joined$Colony.Sample_ID`==names(long)[i]),]

form=part$`joined$Formation.order`[1]

whole=temp[which(temp$`joined$Colony.Sample_ID`!=names(long)[i] & temp$`joined$Formation.order`==form),]

f.test[i]=var.test(log(part$`joined$Area.Zooid`), log(whole$`joined$Area.Zooid`))$p.value

barlett[i]=bartlett.test(list(log(part$`joined$Area.Zooid`), log(whole$`joined$Area.Zooid`)))$p.value

fligner[i]=fligner.test(list(log(part$`joined$Area.Zooid`), log(whole$`joined$Area.Zooid`)))$p.value

}

variance.tested.az=cbind(cbind(f.name, f.test), cbind(fligner, barlett)) #Z1 is ok

#Z2

temp=cbind.data.frame(joined$Area.Ovicell.c, joined$Colony.Sample_ID, joined$Formation.order)

temp=na.omit(temp)

long=which(tapply(temp$`joined$Colony.Sample_ID`, temp$`joined$Colony.Sample_ID`, length)>10)

f.test=NA

barlett=NA

fligner=NA

f.name=NA

#now for each of those in "long", compare variance of the trait with the rest of the data

for (i in 1:length(long)){

f.name[i]=temp[which(temp$`joined$Colony.Sample_ID`==names(long)[i]),]$`joined$Formation.order`[1]

part=temp[which(temp$`joined$Colony.Sample_ID`==names(long)[i]),]

form=part$`joined$Formation.order`[1]

whole=temp[which(temp$`joined$Colony.Sample_ID`!=names(long)[i] & temp$`joined$Formation.order`==form),]

f.test[i]=var.test(log(part$`joined$Area.Ovicell.c`), log(whole$`joined$Area.Ovicell.c`))$p.value

barlett[i]=bartlett.test(list(log(part$`joined$Area.Ovicell.c`), log(whole$`joined$Area.Ovicell.c`)))$p.value

fligner[i]=fligner.test(list(log(part$`joined$Area.Ovicell.c`), log(whole$`joined$Area.Ovicell.c`)))$p.value

}

variance.tested.ovi=cbind(cbind(f.name, f.test), cbind(fligner, barlett)) #Z2 is ok

#Z3

temp=cbind.data.frame(joined$Shape.Zooid, joined$Colony.Sample_ID, joined$Formation.order)

temp=na.omit(temp)

long=which(tapply(temp$`joined$Colony.Sample_ID`, temp$`joined$Colony.Sample_ID`, length)>10)

f.test=NA

barlett=NA

fligner=NA

f.name=NA

#now for each of those in "long", compare variance of the trait with the rest of the data

for (i in 1:length(long)){

f.name[i]=temp[which(temp$`joined$Colony.Sample_ID`==names(long)[i]),]$`joined$Formation.order`[1]

part=temp[which(temp$`joined$Colony.Sample_ID`==names(long)[i]),]

form=part$`joined$Formation.order`[1]

whole=temp[which(temp$`joined$Colony.Sample_ID`!=names(long)[i] & temp$`joined$Formation.order`==form),]

f.test[i]=var.test(log(part$`joined$Shape.Zooid`), log(whole$`joined$Shape.Zooid`))$p.value

barlett[i]=bartlett.test(list(log(part$`joined$Shape.Zooid`), log(whole$`joined$Shape.Zooid`)))$p.value

fligner[i]=fligner.test(list(log(part$`joined$Shape.Zooid`), log(whole$`joined$Shape.Zooid`)))$p.value

}

variance.tested.shape=cbind(cbind(f.name, f.test), cbind(fligner, barlett)) #Z3 is ok except one.

```

**Autozooid area**

```{r, echo=FALSE}

kable(variance.tested.az)

```

**Ovicell area**

```{r, echo=FALSE}

kable(variance.tested.ovi)

```

**Autozooid Shape**

```{r, echo=FALSE}

kable(variance.tested.shape)

```

**Binomial model multivariate N= 169 **

Best model is glm(cbind(f.count,t.count-f.count)~z1+z2+z3+I(z1^2), family=binomial, data=full)

```{r, echo=FALSE}

res=glm(cbind(f.count,t.count-f.count)~z1+z2+z3, family=binomial, data=full)

res1=glm(cbind(f.count,t.count-f.count)~z1+z2+z3+I(z1^2)+I(z2^2)+I(z3^2), family=binomial, data=full)

res2=glm(cbind(f.count,t.count-f.count)~z1+z2+z3+I(z1^2), family=binomial, data=full)

res3=glm(cbind(f.count,t.count-f.count)~z1+z2+z3+I(z2^2), family=binomial, data=full)

res4=glm(cbind(f.count,t.count-f.count)~z1+z2+z3+I(z3^2), family=binomial, data=full)

res5=glm(cbind(f.count,t.count-f.count)~z1+z2+z3+I(z2^2)+I(z3^2), family=binomial, data=full)

res6=glm(cbind(f.count,t.count-f.count)~z1+z2+z3+I(z1^2)+I(z2^2), family=binomial, data=full)

res7=glm(cbind(f.count,t.count-f.count)~z1+z2+z3+I(z1^2)+I(z3^2), family=binomial, data=full)

binom.res=model.sel(res,res1, res2, res3, res4, res5, res6, res7)

kable(binom.res)

#write.csv(file ="res.csv", make.pretty(binom.res))

```

** Best model parameters (binomial glm)**

```{r, echo=FALSE}

summary(res2)

#write.csv(file="res.csv", summary(res2)$coefficients)

```

**Fig. 3**

Solid black lines are predictions and grey are 95% CI for each of the three traits. Note that residual deviance is high.

```{r, echo=FALSE}

postscript(file="Tongima.Fig.3.eps", width=9, height=7, onefile = FALSE, horizontal =FALSE, paper = "special")

par(mfrow=c(1,3), oma=c(3,2,3,0), mar=c(5,4,3,2))

plot(full$z1,full$f.count/full$t.count,xlab="log autozooid area", ylab="ovicells per polymorph", main="", cex.axis=1.4, cex.lab=1.6, pch=20, col="darkgrey", cex=1.5)

title("(a)", line = 0.5, adj=0, cex.main=1.3)

# Do predictions with standard error:

z1.new=seq(min(full$z1),max(full$z1),0.01) # x1 is allowed to vary

z2.new=rep(mean(z2), length(z1.new)) # x2 is constant at mean

z3.new=rep(mean(z3), length(z1.new)) # x3 is constant at mean

newdata=as.data.frame(list(z1=z1.new,z2=z2.new,z3=z3.new))

pred=predict(res2,newdata=newdata,se.fit=TRUE)

# Plot estimate:

lines(newdata$z1, invlogit(pred$fit), lwd=3)

# Plot 95% confidence interval based on normal

# approximation to standard errors

lines(newdata$z1, invlogit(pred$fit-1.96*pred$se.fit),

col="darkgrey", lwd=2)

lines(newdata$z1, invlogit(pred$fit+1.96*pred$se.fit),

col="darkgrey", lwd=2)

plot(full$z2,full$f.count/full$t.count, ylab="", xlab="log ovicell area", main="", cex.axis=1.4, cex.lab=1.6, pch=20, col="darkgrey", cex=1.5)

title("(b)",line = 0.5, adj=0, cex.main=1.3)

z2.new=seq(min(full$z2),max(full$z2),0.01)

z1.new=rep(mean(z1), length(z2.new))

z3.new=rep(mean(z3), length(z2.new))

newdata=as.data.frame(list(z1=z1.new,z2=z2.new,z3=z3.new))

pred=predict(res2,newdata=newdata,se.fit=TRUE)

lines(newdata$z2, invlogit(pred$fit), lwd=3)

lines(newdata$z2, invlogit(pred$fit-1.96*pred$se.fit),

col="darkgrey", lwd=2)

lines(newdata$z2, invlogit(pred$fit+1.96*pred$se.fit),

col="darkgrey", lwd=2)

plot(full$z3,full$f.count/full$t.count, ylab="", xlab="log autozooid shape", main="", cex.axis=1.4, cex.lab=1.6, pch=20, col="darkgrey", cex=1.5)

title("(c)", line = 0.5, adj=0, cex.main=1.3)

z3.new=seq(min(z3), max(z3), 0.01)

z2.new=rep(mean(z2),length(z3.new))

z1.new=rep(mean(z1), length(z3.new))

newdata=as.data.frame(list(z1=z1.new,z2=z2.new,z3=z3.new))

pred=predict(res2,newdata=newdata,se.fit=TRUE)

lines(newdata$z3, invlogit(pred$fit), lwd=3)

lines(newdata$z3, invlogit(pred$fit-1.96*pred$se.fit),

col="darkgrey", lwd=2)

lines(newdata$z3, invlogit(pred$fit+1.96*pred$se.fit),

col="darkgrey", lwd=2)

mtext("Binomial multivariate glm", side=3, outer=T, cex=2, padj=0.5)

dev.off()

```

Diagnostics for best binomial glm (res2 =glm(cbind(f,t-f)~z1+z2+z3+I(z1^2), family=binomial, data=full).

```{r, echo=FALSE}

par(oma=c(1,1,5,1))

model.m.diagnostics <- glm.diag(res2)

glm.diag.plots(res2, model.m.diagnostics)

mtext("Binomial multivariate glm diagnostics", side=3, outer=T, cex=2, padj=0.5)

```

**Univariate interval by interval binomial z1**

**Fig. 4**

```{r, echo=FALSE}

postscript(file="Tongima.Fig.4.eps", width=9, height=7, onefile = FALSE, horizontal =FALSE, paper = "special")

joined.no.na.a=joined[which(!is.na(joined$Area.Zooid)),] #remove NA's N=230

bet=NULL

z.mean=NULL

z.var=NULL

mz1=NULL

coef1=NULL

lab=c("(a)", "(b)", "(c)", "(d)", "(e)", "(f)")

par(mfrow=c(2,3), oma=c(5,3,3,0), mar=c(2,4,2,2))

for (i in 1:length(Formation)){

temp=which(joined.no.na.a$Colony.Form==Formation[i])

temp=joined.no.na.a[temp,]

f=ceiling(temp$Ovi)

t=ceiling(temp$Colony.N_zooids)

z1=log(temp$Area.Zooid)

mz1[i]=mean(z1)

bet[i]=val1

z.mean[i]=mean(log(temp$Area.Zooid)) #original

z.var[i]=sd(log(temp$Area.Zooid))#original std

id=seq(1, length(z1), 1)

id=as.factor(id)

res=glm(cbind(f,t-f)~z1, family=binomial)

val=summary(res)$coefficients

val1=val[2,1]#slope

val2=val[2,2]#slope se

val[2,4]#slope p

sig=val[2,4]<0.05

coef1[i]=val1/val2

plot(z1,f/t,ylab="", xlab="", main=paste(time.labels[i]), ylim=c(0, 0.55), xlim=c(10.8, 11.9), pch=20, col="grey", cex.axis=1.2, cex=1.5)

#text(11,0.5, paste(labels[i], "Ma"), cex=1.3, #col="darkgrey")

if (sig=="TRUE"){

text(11.6,0.45,cex=1.2,paste(round(val1 ,digits=2),"(", round(val2, digits=2), ")*"))

}else{

text(11.6,0.45,cex=1.2,paste(round(val1 ,digits=2),"(", round(val2, digits=2), ")"))

}

title(lab[i], line = 0.5, adj=0, cex.main=1)

z1.new=seq(min(z1),max(z1),0.01)

newdata=as.data.frame(list(z1=z1.new))

pred=predict(res,newdata=newdata,se.fit=TRUE, type="response")

lines(newdata$z1,(pred$fit), lwd=3)

lines(newdata$z1, (pred$fit-1.96*pred$se.fit), lwd=2, col="darkgrey")

lines(newdata$z1, (pred$fit+1.96*pred$se.fit), lwd=2, col="darkgrey")

}

mtext("log autozooid area" , side=1, outer=TRUE, padj=3)

mtext("ovicell per polymorph" , side=2, outer=TRUE, padj=-1)

dev.off()

mtext("binomial univariate" , side=3, outer=TRUE, padj=0)

```

Correlation

```{r, echo=FALSE}

par(mfrow=c(1,1), mar=c(5,4,4,2), oma=c(1,1,1,1))

plot(z.mean , abs(bet) , col="grey", pch=20, cex=2, ylab="strength", xlab="log autozooid area")

x=lm(formula = abs(bet) ~ z.mean )

#abline(x$coefficients[1], x$coefficients[2])

summary(x)

cor.test(abs(bet) , z.mean)

cor.test(abs(bet) , z.mean, method="spearman")

```

**Univariate, interval by interval binomial z2**

This plots log ovicell area as the univariate predictor variable. glm(cbind(f,t-f)~z2, family=binomial) N = 244 (compared with N=169 for full dataset)

```{r, echo=FALSE}

joined.no.na.a=joined[which(!is.na(joined$Area.Ovicell.c)),] #remove NA's N=244

mz2=NULL

coef2=NULL

bet=NULL

z.mean=NULL

z.var=NULL

par(mfrow=c(2,3), oma=c(5,3,3,0), mar=c(2,4,2,2))

for (i in 1:length(Formation)){

temp=which(joined.no.na.a$Colony.Form==Formation[i])

temp=joined.no.na.a[temp,]

f=ceiling(temp$Ovi)

t=ceiling(temp$Colony.N_zooids)

z2=log(temp$Area.Ovicell.c)

mz2[i]=mean(z2)

res=glm(cbind(f,t-f)~z2, family=binomial)

val=summary(res)$coefficients

val1=val[2,1]#slope

val2=val[2,2]#slope se

val[2,4]#slope p

sig=val[2,4]<0.05

coef2[i]=val1/val2

bet[i]=val1

z.mean[i]=mean(log(temp$Area.Ovicell.c)) #original

z.var[i]=sd(log(temp$Area.Ovicell.c))#original std

plot(z2,f/t,ylab="", xlab="log ovicell area", main=paste(time.labels[i]), xlim=c(11.3, 12.2), pch=20, col="grey", ylim=c(0,0.45), cex.axis=1.2, cex=1.5)

#text(11.5,0.38, paste(labels[i], "Ma"), cex=1.3, #col="darkgrey")

if (sig=="TRUE"){

text(11.5, 0.34, cex=1.2,paste(round(val1 ,digits=2),"(", round(val2, digits=2), ")*"))

}else{

text(11.5,0.34,cex=1.2,paste(round(val1 ,digits=2),"(", round(val2, digits=2), ")"))

}

z2.new=seq(min(z2),max(z2),0.01)

newdata=as.data.frame(list(z2=z2.new))

pred=predict(res,newdata=newdata,se.fit=TRUE, type="response")

lines(newdata$z2,(pred$fit), lwd=3)

lines(newdata$z2, (pred$fit-1.96*pred$se.fit), lwd=2, col="darkgrey")

lines(newdata$z2, (pred$fit+1.96*pred$se.fit), lwd=2, col="darkgrey")

}

mtext("log ovicell area" , side=1, outer=TRUE, padj=3)

mtext("ovicell per polymorph " , side=2, outer=TRUE, padj=-1)

mtext("binomial univariate" , side=3, outer=TRUE, padj=0)

```

Correlation

```{r, echo=FALSE}

par(mfrow=c(1,1), mar=c(5,4,4,2), oma=c(1,1,1,1))

plot(z.mean , abs(bet) , col="grey", pch=20, cex=2, ylab="strength", xlab="log ovicell area")

x=lm(formula = abs(bet) ~ z.mean )

#abline(x$coefficients[1], x$coefficients[2])

summary(x)

cor.test(abs(bet) , z.mean)

cor.test(abs(bet) , z.mean, method="spearman")

```

**Univariate, interval by interval binomial z3**

**Fig. 5**

This plots log autozooid shape as the univariate predictor variable. glm(cbind(f,t-f)~z3, family=binomial) N = 230 (compared with N=169 for full dataset)

```{r, echo=FALSE}

postscript(file="Tongima.Fig.5.eps", width=9, height=7, onefile = FALSE, horizontal =FALSE, paper = "special")

joined.no.na.a=joined[which(!is.na(joined$Shape.Zooid)),] #remove NA's N=230

mz3=NULL

coef3=NULL

par(mfrow=c(2,3), oma=c(5,3,3,0), mar=c(2,4,2,2))

bet=NULL

z.mean=NULL

z.var=NULL

for (i in 1:length(Formation)){

temp=which(joined.no.na.a$Colony.Form==Formation[i])

temp=joined.no.na.a[temp,]

f=ceiling(temp$Ovi)

t=ceiling(temp$Colony.N_zooids)

z3=log(temp$Shape.Zooid)

mz3[i]=mean(z3)

res=glm(cbind(f,t-f)~z3, family=binomial)

val=summary(res)$coefficients

val1=val[2,1]#slope

val2=val[2,2]#slope se

val[2,4]#slope p

sig=val[2,4]<0.05

coef3[i]=val1/val2

bet[i]=val1

z.mean[i]=mean(log(temp$Shape.Zooid)) #original

z.var[i]=sd(log(temp$Shape.Zooid))#original std

plot(z3,f/t,ylab=" ", xlab="log autozooid shape", main=paste(time.labels[i]), pch=20, col="grey", ylim=c(0,0.5), xlim=c(0.55,1.15), cex.axis=1.2, cex=1.5)

#text(0.65,0.38, paste(labels[i], "Ma"), cex=1.3, #col="darkgrey")

title(lab[i], line = 0.5, adj=0, cex.main=1)

if (sig=="TRUE"){

text(0.95,0.35,cex=1.2,paste(round(val1 ,digits=2),"(", round(val2, digits=2), ")*"))

}else{

text(0.95,0.35,cex=1.2,paste(round(val1 ,digits=2),"(", round(val2, digits=2), ")"))

}

z3.new=seq(min(z3),max(z3),0.01)

newdata=as.data.frame(list(z3=z3.new))

pred=predict(res,newdata=newdata,se.fit=TRUE, type="response")

lines(newdata$z3,(pred$fit), lwd=3)

lines(newdata$z3, (pred$fit-1.96*pred$se.fit), lwd=2, col="darkgrey")

lines(newdata$z3, (pred$fit+1.96*pred$se.fit), lwd=2, col="darkgrey")

}

mtext("log autozooid shape" , side=1, outer=TRUE, padj=3)

mtext("ovicell per polymorph" , side=2, outer=TRUE, padj=-1)

dev.off()

mtext("binomial univariate" , side=3, outer=TRUE, padj=0)

```

Correlation

```{r, echo=FALSE}

par(mfrow=c(1,1), mar=c(5,4,4,2), oma=c(1,1,1,1))

plot(z.mean , abs(bet) , col="grey", pch=20, cex=2, ylab="strength", xlab="log autozooid shape ")

x=lm(formula = abs(bet) ~ z.mean )

#abline(x$coefficients[1], x$coefficients[2])

summary(x)

cor.test(abs(bet) , z.mean)

cor.test(abs(bet) , z.mean, method="spearman")

```

**Poisson glm for counts of females but use area as covariate in all models**

Here we first use glm of the form

glm(f.count~z1+z2+z3+a, family=poisson(link="log"), data=full) so that area is part of the model. Note that now the f.count is not "standardized", only traits are

The best model is glm(f.count~a+z1+z2+z3+I(z1^2), family=poisson(link="log"), data=full) but glm(f.count~a+z1+z2+z3+I(z1^2)+I(z3^2), family=poisson(link="log"), data=full) is not far behind.

this uses N = 169 and multivariate

This is log link and according to Morssei and Goudie 2016 is ok for interpretation for selection gradient.

```{r, echo=FALSE}

res=glm(f.count~z1+z2+z3+a, family=poisson(link="log"), data=full) #include area as covariate

res1=glm(f.count~a+z1+z2+z3+I(z1^2)+I(z2^2)+I(z3^2), family=poisson(link="log"), data=full)

res2=glm(f.count~a+z1+z2+z3+I(z1^2), family=poisson(link="log"), data=full) #best model

res3=glm(f.count~a+z1+z2+z3+I(z2^2), family=poisson(link="log"), data=full)

res4=glm(f.count~a+z1+z2+z3+I(z3^2), family=poisson(link="log"), data=full)

res5=glm(f.count~a+z1+z2+z3+I(z2^2)+I(z3^2), family=poisson(link="log"), data=full)

res6=glm(f.count~a+z1+z2+z3+I(z1^2)+I(z2^2), family=poisson(link="log"), data=full)

res7=glm(f.count~a+z1+z2+z3+I(z1^2)+I(z3^2), family=poisson(link="log"), data=full)

compare=model.sel(res, res1, res2, res3, res4, res5, res6, res7)

kable(compare)

#write.csv(file="res.csv", compare)

#write.csv(file="res.csv", summary(res2)$coefficients)

#write.csv(file="res.csv", summary(res7)$coefficients)

```

**Estimate from best model**

```{r, echo=FALSE}

kable(summary(res2)$coefficients)

```

**Estimates from second best model**

```{r, echo=FALSE}

kable(summary(res7)$coefficients)

```

**Plot best Poisson glm model f.count given a**

this uses N = 169 and multivariate

```{r, echo=FALSE}

par(mfrow=c(1,3), oma=c(3,1,3,0), mar=c(5,4,3,1))

plot(full$z1,full$f.count/mean(a),ylab="ovicells per unit area", xlab="log autozooid area", main="", cex.axis=1.4, cex.lab=1.6, pch=20, col="darkgrey", cex=1.5, ylim=c(0, 10))

ginv <- res2$family$linkinv

z1.new=seq(min(full$z1),max(full$z1),0.01)

z2.new=rep(mean(full$z2), length(z1.new))

z3.new=rep(mean(full$z3), length(z1.new))

a.new=rep(mean(a), length(z1.new))

newdata=as.data.frame(list(z1=z1.new,z2=z2.new,z3=z3.new, a=a.new))

pred=predict(res2,newdata=newdata,se.fit=TRUE, type="link")

newd <- transform(pred, fitted = ginv(fit), upper = ginv(fit + (2 * se.fit)),

lower = ginv(fit - (2 * se.fit)))

lines(newdata$z1, (newd$fitted)/mean(a), lwd=3)

lines(newdata$z1, (newd$upper)/mean(a),

col="darkgrey", lwd=2)

lines(newdata$z1, newd$lower/mean(a),

col="darkgrey", lwd=2)

title("A.", line = 0.2, adj=0, cex.main=1.8)

#lines(newdata$z1, ginv(pred$fit), lwd=3)

#lines(newdata$z1, ginv(pred$fit-1.96*pred$se.fit),

# col="darkgrey", lwd=2)

#lines(newdata$z1, ginv(pred$fit+1.96*pred$se.fit),

# col="darkgrey", lwd=2)

plot(full$z2,full$f.count/full$a, ylab=" ", xlab="log ovicell area", main="", cex.axis=1.4, cex.lab=1.6, pch=20, col="darkgrey", cex=1.5, ylim=c(0, 9))

z2.new=seq(min(full$z2),max(full$z2),0.01)

z1.new=rep(mean(full$z1), length(z2.new))

z3.new=rep(mean(full$z3), length(z2.new))

a.new=rep(mean(a), length(z2.new))

newdata=as.data.frame(list(z1=z1.new,z2=z2.new,z3=z3.new, a=a.new))

#pred=predict(res2,newdata=newdata,se.fit=TRUE, type="response")

pred=predict(res2,newdata=newdata,se.fit=TRUE, type="link")

newd <- transform(pred, fitted = ginv(fit), upper = ginv(fit + (2 * se.fit)),

lower = ginv(fit - (2 * se.fit)))

lines(newdata$z2, (newd$fitted)/mean(a), lwd=3)

lines(newdata$z2, (newd$upper)/mean(a),

col="darkgrey", lwd=2)

lines(newdata$z2, newd$lower/mean(a),

col="darkgrey", lwd=2)

title("B.", line = 0.2, adj=0, cex.main=1.8)

#lines(newdata$z2, (pred$fit/mean(a)), lwd=3)

#lines(newdata$z2, (pred$fit/mean(a)-1.96*pred$se.fit),

# col="darkgrey", lwd=2)

#lines(newdata$z2, (pred$fit/mean(a)+1.96*pred$se.fit),

# col="darkgrey", lwd=2)

plot(full$z3,full$f.count/full$a, ylab=" ", xlab="log autozooid shape",main="", cex.axis=1.4, cex.lab=1.6, pch=20, col="darkgrey", cex=1.5, ylim=c(0, 9))

z3.new=seq(min(full$z3), max(full$z3), 0.01)

z2.new=rep(mean(full$z2),length(z3.new))

z1.new=rep(mean(full$z1), length(z3.new))

a.new=rep(mean(a), length(z3.new))

newdata=as.data.frame(list(z1=z1.new,z2=z2.new,z3=z3.new, a=a.new))

#pred=predict(res2,newdata=newdata,se.fit=TRUE, type="response")

pred=predict(res2,newdata=newdata,se.fit=TRUE, type="link")

newd <- transform(pred, fitted = ginv(fit), upper = ginv(fit + (2 * se.fit)),

lower = ginv(fit - (2 * se.fit)))

lines(newdata$z3, (newd$fitted)/mean(a), lwd=3)

lines(newdata$z3, (newd$upper)/mean(a),

col="darkgrey", lwd=2)

lines(newdata$z3, newd$lower/mean(a),

col="darkgrey", lwd=2)

title("C.", line = 0.2, adj=0, cex.main=1.8)

#lines(newdata$z3, (pred$fit/mean(a)), lwd=3)

#lines(newdata$z3, (pred$fit/mean(a)-1.96*pred$se.fit),

# col="darkgrey", lwd=2)

#lines(newdata$z3, (pred$fit/mean(a)+1.96*pred$se.fit),

# col="darkgrey", lwd=2)

mtext("Poisson multivariate glm", side=3, outer=T, cex=2, padj=0.5)

```

**Diagnostics for best poisson glm**

Looks worse than lm.

```{r, echo=FALSE}

par(oma=c(2,2,5,2))

model.m.diagnostics <- glm.diag(res2)

glm.diag.plots(res2, model.m.diagnostics)

mtext("Poisson GLM diagnostics", side=3, outer=T, cex=1.5, padj=0.5)

```

**Univariate poisson models: include all data now**

**linear z1**

```{r, echo=FALSE}

joined.no.na.a=joined[which(!is.na(joined$Area.Zooid)),] #remove NA's, N= 230

bet=NULL

z.mean=NULL

z.var=NULL

par(mfrow=c(2,3), oma=c(5,3,3,0), mar=c(2,4,2,2))

for (i in 1:length(Formation)){

temp=which(joined.no.na.a$Colony.Form==Formation[i])

temp=joined.no.na.a[temp,]

f=temp$Ovi

f.count=ceiling(temp$Colony.Ovi)

z1=log(temp$Area.Zooid)/mean(log(temp$Area.Zooid))

a=temp$Colony.area

res=glm(f.count~z1+a, family=poisson(link="log"), data=temp)

val=summary(res)$coefficients

val1=val[2,1]#slope

val2=val[2,2]#slope se

val[2,4]#slope p

sig=val[2,4]<0.05

#print(summary(res))

bet[i]=val1

z.mean[i]=mean(log(temp$Area.Zooid)) #original

z.var[i]=sd(log(temp$Area.Zooid))#original std

plot(z1,f.count/a,ylab="", xlab="", main=paste(time.labels[i]), pch=20, col="grey", cex.axis=1.2, cex=1.5, xlim=c(0.965, 1.03), ylim=c(0,7))

text(11,0.5, labels[i], cex=1.3, col="darkgrey")

if (sig=="TRUE"){

text(0.99,6,cex=1.2,paste(round(val1 ,digits=2),"(", round(val2, digits=2), ")*"))

}else{

text(0.99,6,cex=1.2,paste(round(val1 ,digits=2),"(", round(val2, digits=2), ")"))

}

z1.new=seq(min(z1),max(z1),0.001)

a.new=rep(mean(a),length(z1.new))

newdata=as.data.frame(list(z1=z1.new, a=a.new))

#pred=predict(res,newdata=newdata,se.fit=TRUE, type="response")

pred=predict(res,newdata=newdata,se.fit=TRUE, type="link")

newd <- transform(pred, fitted = ginv(fit), upper = ginv(fit + (2 * se.fit)),

lower = ginv(fit - (2 * se.fit)))

lines(newdata$z1, (newd$fitted)/mean(a), lwd=3)

lines(newdata$z1, (newd$upper)/mean(a),

col="darkgrey", lwd=2)

lines(newdata$z1, newd$lower/mean(a),

col="darkgrey", lwd=2)

# Plot estimate:

#lines(newdata$z1,(pred$fit/mean(a)), lwd=3)

#lines(newdata$z1, (pred$fit/mean(a)-1.96*pred$se.fit), lwd=2, col="darkgrey")

#lines(newdata$z1, (pred$fit/mean(a)+1.96*pred$se.fit), lwd=2, col="darkgrey")

}

mtext("log autozooid area" , side=1, outer=TRUE, cex=1.8, line=2)

mtext("ovicell per unit area" , side=2, outer=TRUE, cex=1.8)

mtext("Poisson univariate glm" , side=3, outer=TRUE, cex=1.5)

```

**No relationship between z1 mean and strength of selection**

```{r, echo=FALSE}

par(mfrow=c(1,1), mar=c(5,4,4,2), oma=c(1,1,1,1))

plot(z.mean , abs(bet) , col="grey", pch=20, cex=2, xlab="log autozooid area", ylab="strength")

x=lm(formula = abs(bet) ~ z.mean )

#abline(x$coefficients[1], x$coefficients[2])

summary(x)

cor.test(z.mean , abs(bet))

cor.test(z.mean , abs(bet), method="spearman")

```

**Univariate poisson models: include all data now z2**

```{r, echo=FALSE}

joined.no.na.a=joined[which(!is.na(joined$Area.Ovicell.c)),] #remove NA's N = 244

bet=NULL

z.mean=NULL

z.var=NULL

par(mfrow=c(2,3), oma=c(5,3,3,0), mar=c(2,4,2,2))

for (i in 1:length(Formation)){

temp=which(joined.no.na.a$Colony.Form==Formation[i])

temp=joined.no.na.a[temp,]

f=temp$Ovi

f.count=ceiling(temp$Colony.Ovi)

z2=log(temp$Area.Ovicell.c)/mean(log(temp$Area.Ovicell.c))

a=temp$Colony.area

res=glm(f.count~z2+a, family=poisson(link="log"), data=temp)

val=summary(res)$coefficients

val1=val[2,1]#slope

val2=val[2,2]#slope se

val[2,4]#slope p

sig=val[2,4]<0.05

#print(summary(res))

bet[i]=val1

z.mean[i]=mean(log(temp$Area.Ovicell.c)) #original

z.var[i]=sd(log(temp$Area.Ovicell.c))#original std

plot(z2,f.count/a,ylab="", xlab="", main=paste(time.labels[i]), pch=20, col="grey", cex.axis=1.2, cex=1.5, ylim=c(0,7), xlim=c(0.96, 1.035))

text(11,0.5, labels[i], cex=1.3, col="darkgrey")

if (sig=="TRUE"){

text(0.99,6,cex=1.2,paste(round(val1 ,digits=2),"(", round(val2, digits=2), ")*"))

}else{

text(0.99,6,cex=1.2,paste(round(val1 ,digits=2),"(", round(val2, digits=2), ")"))

}

z2.new=seq(min(z2),max(z2),0.001)

a.new=rep(mean(a),length(z2.new))

newdata=as.data.frame(list(z2=z2.new, a=a.new))

#pred=predict(res,newdata=newdata,se.fit=TRUE, type="response")

pred=predict(res,newdata=newdata,se.fit=TRUE, type="link")

newd <- transform(pred, fitted = ginv(fit), upper = ginv(fit + (2 * se.fit)),

lower = ginv(fit - (2 * se.fit)))

lines(newdata$z2, (newd$fitted)/mean(a), lwd=3)

lines(newdata$z2, (newd$upper)/mean(a),

col="darkgrey", lwd=2)

lines(newdata$z2, newd$lower/mean(a),

col="darkgrey", lwd=2)

# Plot estimate:

#lines(newdata$z2,(pred$fit/mean(a)), lwd=3)

#lines(newdata$z2, (pred$fit/mean(a)-1.96*pred$se.fit), lwd=2, col="darkgrey")

#lines(newdata$z2, (pred$fit/mean(a)+1.96*pred$se.fit), lwd=2, col="darkgrey")

}

mtext("log ovicell area" , side=1, outer=TRUE, cex=1.8, line=2)

mtext("ovicell per unit area" , side=2, outer=TRUE, cex=1.8)

mtext("Poisson univariate" , side=3, outer=TRUE, cex=1.5)

```

**No relationship between mean z2 and strength of "selection"**

```{r, echo=FALSE}

par(mfrow=c(1,1), mar=c(5,4,4,2), oma=c(1,1,1,1))

plot(z.mean , abs(bet) , col="grey", pch=20, cex=2, ylab="strength", xlab="log ovicell area")

x=lm(formula = abs(bet) ~ z.mean )

#abline(x$coefficients[1], x$coefficients[2])

summary(x)

cor.test(abs(bet) , z.mean)

cor.test(abs(bet) , z.mean, method="spearman")

```

**Univariate models: linear z3**

```{r, echo=FALSE}

joined.no.na.a=joined[which(!is.na(joined$Shape.Zooid)),] #remove NA's N = 230

bet=NULL

z.mean=NULL

z.var=NULL

par(mfrow=c(2,3), oma=c(5,3,3,0), mar=c(2,4,2,2))

for (i in 1:length(Formation)){

temp=which(joined.no.na.a$Colony.Form==Formation[i])

temp=joined.no.na.a[temp,]

f=temp$Ovi

f.count=ceiling(temp$Colony.Ovi)

z3=log(temp$Shape.Zooid)/mean(log(temp$Shape.Zooid))

a=temp$Colony.area

res=glm(f.count~z3+a, family=poisson(link="log"), data=temp)

val=summary(res)$coefficients

val1=val[2,1]#slope

val2=val[2,2]#slope se

val[2,4]#slope p

sig=val[2,4]<0.05

#print(summary(res))

bet[i]=val1

z.mean[i]=mean(log(temp$Shape.Zooid)) #original

z.var[i]=sd(log(temp$Shape.Zooid))#original std

plot(z3,f.count/a,ylab="", xlab="", main=paste(time.labels[i]), pch=20, col="grey", cex.axis=1.2, cex=1.5, xlim=c(0.65, 1.35), ylim=c(0,7))

text(11,0.5, labels[i], cex=1.3, col="darkgrey")

if (sig=="TRUE"){

text(0.85,6,cex=1.2,paste(round(val1 ,digits=2),"(", round(val2, digits=2), ")*"))

}else{

text(0.85,6,cex=1.2,paste(round(val1 ,digits=2),"(", round(val2, digits=2), ")"))

}

z3.new=seq(min(z3),max(z3),0.001)

a.new=rep(mean(a),length(z3.new))

newdata=as.data.frame(list(z3=z3.new, a=a.new))

#pred=predict(res,newdata=newdata,se.fit=TRUE, type="response")

pred=predict(res,newdata=newdata,se.fit=TRUE, type="link")

newd <- transform(pred, fitted = ginv(fit), upper = ginv(fit + (2 * se.fit)),

lower = ginv(fit - (2 * se.fit)))

lines(newdata$z3, (newd$fitted)/mean(a), lwd=3)

lines(newdata$z3, (newd$upper)/mean(a),

col="darkgrey", lwd=2)

lines(newdata$z3, newd$lower/mean(a),

col="darkgrey", lwd=2)

# Plot estimate:

#lines(newdata$z3,(pred$fit/mean(a)), lwd=3)

#lines(newdata$z3, (pred$fit/mean(a)-1.96*pred$se.fit/mean(a)), lwd=2, #col="darkgrey")

#lines(newdata$z3, (pred$fit/mean(a)+1.96*pred$se.fit/mean(a)), lwd=2, col="darkgrey")

}

mtext("log autozooid shape" , side=1, outer=TRUE, cex=1.8, line=2)

mtext("ovicell per unit area" , side=2, outer=TRUE, cex=1.8)

mtext("Poisson univariate" , side=3, outer=TRUE, cex=1.5)

```

**No relationship between mean z3 and strength of "selection"**

```{r, echo=FALSE}

par(mfrow=c(1,1), mar=c(5,4,4,2), oma=c(1,1,1,1))

plot(z.mean , abs(bet) , col="grey", pch=20, cex=2, ylab="strength", xlab="log autozooid shape")

x=lm(formula = abs(bet) ~ z.mean )

#abline(x$coefficients[1], x$coefficients[2])

summary(x)

cor.test(abs(bet) , z.mean)

cor.test(abs(bet) , z.mean, method="spearman")

```

**Fecundity and ovicell area**

```{r, echo=FALSE}

par(mfrow=c(2,1), mar=c(4,5,1,2), oma=c(2,0,2,0))

plot(joined$Colony.Fec1, log(joined$Area.Ovicell.c), ylab="log ovicell area", xlab="Ovicells per unit area", col="grey", pch=20, cex=1.5)

val=lm(log(joined$Area.Ovicell.c)~joined$Colony.Fec1)

abline(val$coefficients[1],val$coefficients[2], lwd=2)

plot(joined$Colony.Fec2, log(joined$Area.Ovicell.c), ylab="log ovicell area ", xlab="Ovicells per polymorph", col="grey", pch=20, cex=1.5)

val=lm(log(joined$Area.Ovicell.c)~joined$Colony.Fec2)

abline(val$coefficients[1],val$coefficients[2], lwd=2)

```

**Models for traits and fecundity with O18 and ecological interactions as explanatory variables**

Note that

interactions = intra + inter specific overgrowth observed

self.interactions = intraspecific overgrwoth

other = inter specific overgrwoth

onShell = overcrowding (i.e. any other bryozoans on that particular shell)

Add https://lorraine-lisiecki.com/LR04stack.txt

```{r,echo=FALSE}

#join the data

int.0=subset(int, select=c("Unique.Colony", "interactions", "self.interactions", "onShell","other", "Formation_name", "Formation.order"))

joined=join(joined,int.0, by="Unique.Colony", type="right")

joined$M=ceiling(joined$Colony.reproductive-joined$Colony.Ovi)#

joined$R=ceiling(joined$Colony.Ovi)#

joined$N=ceiling(joined$Colony.N_zooids)

joined$A=ceiling(joined$Colony.N_autozooids)

joined=(na.omit(joined))

```

**Table S Model comparison for autozooid size **

```{r, echo=FALSE}

oxygen.18= read.csv("O18.csv")

bottom=c(2290, 2030, 920, 680, 430, 330)

top=c(2080, 1970, 900, 620, 400, 300)

ave.O18=NULL

sd.ave.O18=NULL

for (i in 1:6)

{temp=oxygen.18$d18O[which(oxygen.18$Time<bottom[i] & oxygen.18$Time>top[i])]

ave.O18[i]=median(temp)

sd.ave.O18[i]=sd(temp)

}

O18=cbind(cbind(ave.O18, sd.ave.O18), cbind(bottom, top))

O18=as.data.frame(O18)

rownames(O18)=Formation

joined$density=joined$Colony.N_zooids/joined$Colony.area

joined$O18=NA

joined$O18.sd=NA

for (i in 1:dim(joined)[1])

{index=which(rownames(O18) ==joined$Formation_name[i])

joined$O18[i]=O18$ave.O18[index]

joined$O18.sd[i]=O18$sd.ave.O18[index]

}

full.model=lm(formula = log(Area.Zooid) ~ self.interactions+other+onShell+O18+O18.sd+log(Shape.Zooid)+log(Area.Ovicell.c),data=joined)

oxy=lm(formula = log(Area.Zooid) ~ O18+O18.sd, data=joined)

traits=lm(formula = log(Area.Zooid) ~ log(Shape.Zooid)+log(Area.Ovicell.c), data=joined)

traits.oxy=lm(formula = log(Area.Zooid) ~ log(Shape.Zooid)+log(Area.Ovicell.c)+O18+O18.sd, data=joined)

ecol=lm(formula = log(Area.Zooid) ~ self.interactions+other+onShell, ,data=joined)

ecol.oxy=lm(formula = log(Area.Zooid) ~ O18+O18.sd+self.interactions+other+onShell, data=joined)

x=model.sel(full.model, oxy, traits, ecol, traits.oxy, ecol.oxy)

kable(x)

#write.csv(make.pretty(x), "res.csv")

#write.csv(summary(full.model)$coefficients, "res.csv")

summary(full.model)

summary(traits.oxy)

```

**Plot O18 effect for autozooid size while controlling for other factors in full.model**

Fig. 6

```{r, echo=FALSE}

postscript(file="Tongima.Fig.6.eps", width=9, height=7, onefile = FALSE, horizontal =FALSE, paper = "special")

O18.new=seq(min(joined$O18), max(joined$O18), 0.1)

self.new=rep(mean(joined$self.interactions), length(O18.new))

other.new=rep(mean(joined$other), length(O18.new))

onShell.new=rep(mean(joined$onShell), length(O18.new))

O18.sd.new=rep(mean(joined$O18.sd), length(O18.new))

shape.new=rep(((mean(joined$Shape.Zooid))), length(O18.new))

ovi.new=rep((mean(joined$Area.Ovicell.c)), length(O18.new))

newdata=as.data.frame(list(O18=O18.new, self.interactions =self.new, other=other.new,onShell=onShell.new, O18.sd= O18.sd.new, Shape.Zooid=shape.new, Area.Ovicell.c=ovi.new))

par(mfrow=c(1,1), mar=c(5,5,4,2), oma=c(0,0,0,0))

plot(jitter(joined$O18), log(joined $Area.Zooid), pch=20, cex=2, col="grey" ,ylab="log autozooid size",

xlab=expression(paste(delta, "O18")), cex.lab=1.8, cex.axis=1.5)

oxygen=tapply(log(joined $Area.Zooid), as.factor(joined$O18), mean)

o18.t=as.numeric(names(oxygen))

points(o18.t, oxygen, cex=2.5, pch=20)

pred=predict(full.model,newdata=newdata,se.fit=TRUE, type="response")

lines(newdata$O18, (pred$fit), lwd=3)

lines(newdata$O18, (pred$fit-1.96*pred$se.fit),

col="darkgrey", lwd=2)

lines(newdata$O18, (pred$fit+1.96*pred$se.fit),

col="darkgrey", lwd=2)

dev.off()

```

**Table S Model comparison for autozooid shape**

```{r, echo=FALSE}

full.model=lm(formula = log(Shape.Zooid) ~ self.interactions+other+onShell+O18+O18.sd+log(Area.Zooid)+log(Area.Ovicell.c),data=joined)

oxy=lm(formula =log(Shape.Zooid) ~ O18+O18.sd, data=joined)

traits=lm(formula = log(Shape.Zooid) ~ log(Area.Zooid)+log(Area.Ovicell.c), data=joined)

traits.oxy=lm(formula = log(Shape.Zooid) ~ log(Area.Zooid)+log(Area.Ovicell.c)+O18+O18.sd, data=joined)

ecol=lm(formula = log(Shape.Zooid) ~ self.interactions+other+onShell, ,data=joined)

ecol.oxy=lm(formula = log(Shape.Zooid) ~ O18+O18.sd+self.interactions+other+onShell, data=joined)

x=model.sel(full.model, oxy, traits, ecol, traits.oxy, ecol.oxy)

kable(x)

#write.csv(make.pretty(x), "res.csv")

#write.csv(summary(full.model)$coefficients, "res.csv")

summary(full.model)

```

**Table S Model comparison for ovicell size **

```{r, echo=FALSE}

full.model=lm(formula = log(Area.Ovicell.c)~ self.interactions+other+onShell+O18+O18.sd+log(Area.Zooid)+log(Shape.Zooid),data=joined)

oxy=lm(formula =log(Area.Ovicell.c) ~ O18+O18.sd, data=joined)

traits=lm(formula = log(Area.Ovicell.c) ~ log(Area.Zooid)+log(Shape.Zooid), data=joined)

traits.oxy=lm(formula = log(Area.Ovicell.c) ~ log(Area.Zooid)+log(Shape.Zooid)+O18+O18.sd, data=joined)

ecol=lm(formula = log(Area.Ovicell.c) ~ self.interactions+other+onShell, ,data=joined)

ecol.oxy=lm(formula = log(Area.Ovicell.c) ~ O18+O18.sd+self.interactions+other+onShell, data=joined)

x=model.sel(full.model, oxy, traits, ecol, traits.oxy, ecol.oxy)

kable(x)

#write.csv(make.pretty(x), "res.csv")

#write.csv(summary(full.model)$coefficients, "res.csv")

summary(full.model)

```

**for fecundity (binomial)**

```{r, echo=FALSE}

full.model=glm(formula = cbind(R,N-R)~ self.interactions+other+onShell+O18+O18.sd+log(Area.Zooid)+log(Shape.Zooid),log(Area.Ovicell.c), data=joined,family=binomial)

oxy=glm(formula = cbind(R,N-R)~ O18+O18.sd, data=joined,family=binomial)

traits=glm(formula = cbind(R,N-R)~ log(Area.Zooid)+log(Shape.Zooid)+log(Area.Ovicell.c), data=joined,family=binomial)

traits.oxy=glm(formula = cbind(R,N-R)~log(Area.Zooid)+log(Shape.Zooid)+log(Area.Ovicell.c)+O18+O18.sd, data=joined,family=binomial)

ecol=glm(formula = cbind(R,N-R)~self.interactions+other+onShell, ,data=joined,family=binomial)

#ecol.1=lm(formula = log(Area.Ovicell.c)~ interactions+onShell, data=joined)

ecol.oxy=glm(formula = cbind(R,N-R)~ O18+O18.sd+self.interactions+other+onShell, data=joined,family=binomial)

x=model.sel(full.model, oxy, traits, ecol, traits.oxy, ecol.oxy)

kable(x)

#write.csv(make.pretty(x), "res.csv")

#write.csv(summary(traits.oxy)$coefficients, "res.csv")

summary(traits.oxy)

```

**Plot O18 and O18 sd effect for fecundity while controlling for other factors in traits.oxy model**

```{r, echo=FALSE}

par(mfrow=c(1,2))

O18.new=seq(min(joined$O18), max(joined$O18), 0.1)

O18.sd.new=rep(mean(joined$O18.sd), length(O18.new))

log.Area.Ovicell.c.new=rep(mean((joined$Area.Ovicell.c)), length(O18.new))

log.Shape.Zooid.new=rep(mean((joined$Shape.Zooid)), length(O18.new))

log.Area.Zooid=rep(mean((joined$Area.Zooid)), length(O18.new))

newdata=as.data.frame(list(O18=O18.new, O18.sd= O18.sd.new,Area.Zooid=log.Area.Zooid, Shape.Zooid=log.Shape.Zooid.new, Area.Ovicell.c=log.Area.Ovicell.c.new))

#par(mfrow=c(1,1), mar=c(5,5,4,2), oma=c(0,0,0,0))

plot(jitter(joined$O18), joined$R/(joined$N-joined$R), pch=20, cex=2, col="grey" ,ylab="ovicells per polymorph",

xlab=expression(paste(delta, "O18")), cex.lab=1.8, cex.axis=1.5)

oxygen=tapply(joined$R/(joined$N-joined$R), as.factor(joined$O18), mean)

o18.t=as.numeric(names(oxygen))

points(o18.t, oxygen, cex=2.5, pch=20)

invlogit=function(x) 1/(1+exp(-x))

pred=predict(traits.oxy,newdata=newdata,se.fit=TRUE)

lines(newdata$O18, invlogit(pred$fit), lwd=3)

lines(newdata$O18, invlogit(pred$fit-1.96*pred$se.fit),

col="darkgrey", lwd=2)

lines(newdata$O18, invlogit(pred$fit+1.96*pred$se.fit),

col="darkgrey", lwd=2)

O18.sd.new=seq(min(joined$O18.sd), max(joined$O18.sd), 0.01)

O18.new=rep(mean(joined$O18), length(O18.sd.new))

log.Area.Ovicell.c.new=rep(mean((joined$Area.Ovicell.c)), length(O18.new))

log.Shape.Zooid.new=rep(mean((joined$Shape.Zooid)), length(O18.new))

log.Area.Zooid=rep(mean((joined$Area.Zooid)), length(O18.new))

newdata=as.data.frame(list(O18=O18.new, O18.sd= O18.sd.new,Area.Zooid=log.Area.Zooid, Shape.Zooid=log.Shape.Zooid.new, Area.Ovicell.c=log.Area.Ovicell.c.new))

#par(mfrow=c(1,1), mar=c(5,5,4,2), oma=c(0,0,0,0))

plot(jitter(joined$O18.sd), joined$R/(joined$N-joined$R), pch=20, cex=2, col="grey" ,ylab="ovicells per polymorph",

xlab=expression(paste(delta, "O18 sd")), cex.lab=1.8, cex.axis=1.5)

oxygen=tapply(joined$R/(joined$N-joined$R), as.factor(joined$O18.sd), mean)

o18.t=as.numeric(names(oxygen))

points(o18.t, oxygen, cex=2.5, pch=20)

invlogit=function(x) 1/(1+exp(-x))

pred=predict(traits.oxy,newdata=newdata,se.fit=TRUE)

lines(newdata$O18.sd, invlogit(pred$fit), lwd=3)

lines(newdata$O18.sd, invlogit(pred$fit-1.96*pred$se.fit),

col="darkgrey", lwd=2)

lines(newdata$O18.sd, invlogit(pred$fit+1.96*pred$se.fit),

col="darkgrey", lwd=2)

plot(allEffects(traits.oxy))

```

**Fig.S Two measures of Ovicell area are highly correlated**

```{r, echo=FALSE}

# Circle and area measures no difference

par(mfrow=c(1,1),mar=c(5,4,4,2))

plot(Measurements$Ovicell.area, Measurements$Ovicell.circle, ylab="ovicell circle area", xlab="ovicell L times W", pch=20, col="grey")

val=summary(lm(Measurements$Ovicell.circle~ Measurements$Ovicell.area))#Adjusted R-squared: 0.9883

abline(val$coefficients[1,1], val$coefficients[2,1], col="blue", lwd=3)

legend("topleft", paste("R.sq =", round(val$r.squared, digits =3), pch=""), bty="n")

```

**Fig. S Repeatibility of size measurments is high**

```{r, echo=FALSE}

Repeated= read.xlsx("Antarctothoa_dataset_07.08.2019.xlsx", 4)

Repeated$Ovi.area1=Repeated$Ovicell_length_1*Repeated$Ovicell_width_1

Repeated$Ovi.area2=Repeated$Ovicell_length_2*Repeated$Ovicell_width_2

Repeated$Ovi.area3=Repeated$Ovicell_length_3*Repeated$Ovicell_width_3

par(mfrow=c(2,3), mar=c(4,4,2,2), oma=c(1,1,3,1))

plot(Repeated$Ovi.area1, Repeated$Ovi.area2, ylab="Ovicell Area 1", xlab="Ovicell Area 2", pch=20, col="grey")

abline(0,1,col="red")

val=summary(lm(formula = Repeated$Ovi.area1 ~ Repeated$Ovi.area2))$r.sq #r.sq= 0.9738

legend("topleft", paste("R.sq = ", round(val, digits=3)), pch= " ", bty="n", cex=1.8)

plot(Repeated$Ovi.area2, Repeated$Ovi.area3, ylab="Ovicell Area 2", xlab="Ovicell Area 3", pch=20, col="grey")

abline(0,1,col="red")

val=summary(lm(formula = Repeated$Ovi.area2 ~ Repeated$Ovi.area3))$r.sq# r.sq = 0.9699

legend("topleft", paste("R.sq = ", round(val, digits=3)), pch= " ", bty="n", cex=1.8)

plot(Repeated$Ovi.area1, Repeated$Ovi.area3, ylab="Ovicell Area 1", xlab="Ovicell Area 3", pch=20, col="grey")

val=summary(lm(formula = Repeated$Ovi.area1 ~ Repeated$Ovi.area3))$r.sq #r.sq= 0.9741

abline(0,1,col="red")

legend("topleft", paste("R.sq = ", round(val, digits=3)), pch= " ", bty="n", cex=1.8)

Repeated$Zooid.area1=Repeated$Zooid_length_1*Repeated$Zooid_width_1

Repeated$Zooid.area2=Repeated$Zooid_length_2*Repeated$Zooid_width_2

Repeated$Zooid.area3=Repeated$Zooid_length_3*Repeated$Zooid_width_3

plot(Repeated$Zooid.area1, Repeated$Zooid.area2, ylab="Autozooid Area 1", xlab="Autozooid Area 2", pch=20, col="grey")

abline(0,1,col="red")

val=summary(lm(formula = Repeated$Zooid.area1 ~ Repeated$Zooid.area2))$r.sq #r.sq= 0.9931

abline(0,1,col="red")

legend("topleft", paste("R.sq = ", round(val, digits=3)), pch= " ", bty="n", cex=1.8)

plot(Repeated$Zooid.area2, Repeated$Zooid.area3, ylab="Autozooid Area 2", xlab="Autozooid Area 3", pch=20, col="grey")

abline(0,1,col="red")

val=summary(lm(formula = Repeated$Zooid.area2 ~ Repeated$Zooid.area3))$r.sq# r.sq = 0.9915

abline(0,1,col="red")

legend("topleft", paste("R.sq = ", round(val, digits=3)), pch= " ", bty="n", cex=1.8)

plot(Repeated$Zooid.area1, Repeated$Zooid.area3 , ylab="Autozooid Area 1", xlab="Autozooid Area 3", pch=20, col="grey")

val=summary(lm(formula = Repeated$Zooid.area1 ~ Repeated$Zooid.area3))$r.sq #r.sq=0.9941

abline(0,1,col="red")

legend("topleft", paste("R.sq = ", round(val, digits=3)), pch= " ", bty="n", cex=1.8)

```

**Fig. S Autozooid length and width both correlate with Area but not each other**

```{r, echo=FALSE}

# L and W do little to predict each other, Adjusted R-squared: 0.1015

par(mfrow=c(3,1), mar=c(4,6,2,2))

plot( Measurements$Zooid_width, Measurements$Zooid_length, xlab="Autozooid width", ylab="Autozooid length", cex.axis=1.5, cex.lab=1.8, col="grey", pch=20)

val=summary(lm(Measurements$Zooid_length ~ Measurements$Zooid_width ))

abline(val$coefficients[1,1], val$coefficients[2,1], col="blue", lwd=2)

legend("topleft", paste("R.sq =", round(val$r.squared, digits =3), pch=""), bty="n", cex=1.8, col="grey", pch=20)

# L and W are much better at predicting area

plot( Measurements$Zooid.area, Measurements$Zooid_width, xlab="Autozooid area", ylab="Autozooid width", cex.axis=1.5, cex.lab=1.8, col="grey", pch=20)

val=summary(lm(Measurements$Zooid_width ~ Measurements$Zooid.area))#Adjusted R-squared: 0.7192

abline(val$coefficients[1,1], val$coefficients[2,1], col="blue", lwd=2)

legend("topleft", paste("R.sq =", round(val$r.squared, digits =3), pch=""), bty="n", cex=1.8, col="grey", pch=20)

plot( Measurements$Zooid.area, Measurements$Zooid_length, xlab="Autozooid area", ylab="Autozooid length", cex.axis=1.5, cex.lab=1.8, col="grey", pch=20)

val=summary(lm(Measurements$Zooid_length ~ Measurements$Zooid.area))#Adjusted R-squared: 0.5862

legend("topleft", paste("R.sq =", round(val$r.squared, digits =3), pch=""), bty="n", cex=1.8, col="grey", pch=20)

abline(val$coefficients[1,1], val$coefficients[2,1], col="blue", lwd=2)

```

**Fig. S. Variation in fecundity (females per area) in sampling units** DELETE??

Fec1= number of ovicells per area (used to estimate wa)

```{r, echo=FALSE}

par(mfrow=c(2,2), oma = c(0, 2, 2, 0), mar=c(3,3,3,2))

hist(tapply(Data$Fec1, Data$Image_ID, mean, na.rm=TRUE), main="Spot", cex.main=1, xlab="", ylab="", col="grey")

hist(tapply(Data$Fec1, Data$Unique.Colony, mean, na.rm=TRUE), main="Colony", cex.main=1, xlab="", ylab="", col="grey")

hist(tapply(Data$Fec1, Data$Unique.Shell, mean, na.rm=TRUE), main="Shell", cex.main=1, xlab="", ylab="", col="grey")

hist(tapply(Data$Fec1, Data$Sample_ID, mean, na.rm=TRUE), main="Sample",cex.main=1, xlab="", ylab="", col="grey")

mtext("Average Fecundity (Females per Area)", outer = TRUE, cex = 1, font=4, side =3 )

mtext("Number", outer = TRUE, cex = 1, font=4, side =2)

```

**Fig. S. Variation in fecundity (females per polymorph) in sampling units** DELETE??

Fec2= number of ovicells per polymorph

```{r, echo=FALSE}

par(mfrow=c(2,2), oma = c(0, 2, 2, 0), mar=c(3,3,3,2))

hist(tapply(Data$Fec2, Data$Image_ID, mean, na.rm=TRUE), main="Spot", cex.main=1, xlab="", ylab="", col="grey")

hist(tapply(Data$Fec2, Data$Unique.Colony, mean, na.rm=TRUE), main="Colony", cex.main=1, xlab="", ylab="", col="grey")

hist(tapply(Data$Fec2, Data$Unique.Shell, mean, na.rm=TRUE), main="Shell", cex.main=1, xlab="", ylab="", col="grey")

hist(tapply(Data$Fec2, Data$Sample_ID, mean, na.rm=TRUE), main="Sample",cex.main=1, xlab="", ylab="", col="grey")

mtext("Average Fecundity (Females per polymorph)", outer = TRUE, cex = 1, font=4, side =3 )

mtext("Number", outer = TRUE, cex = 1, font=4, side =2)

```

**compare autozooid shape and area** DELETE?

```{r, echo=FALSE}

par(mfrow=c(1,1), mar=c(5,5,2,2), oma=c(0,0,0,0))

plot(log(joined$Area.Zooid), (joined$Shape.Zooid), ylab="Shape", xlab="Area", pch=20, col="grey")

val=lm((joined$Shape.Zooid)~log(joined$Area.Zooid))

abline(val$coefficients[1],val$coefficients[2], col="blue", lwd=2)

```

**Fig. S. Diffrent measures of fecundity are highly correlated**

```{r, echo=FALSE}

par(mfrow=c(1,1), mar=c(5,5,2,2), oma=c(0,0,0,0))

plot(Data$Fec1, Data$Fec2, ylab="ovicells per polymorph", xlab="ovicells per unit area", pch=20, col="grey")

val=lm((Data$Fec2~Data$Fec1))

abline(val$coefficients[1],val$coefficients[2], col="blue", lwd=2)

text(2, 0.5, "r.sq=0.95")

```

##"plasticity"##

**Compare within colony variance of traits with all data or within formation data**

In the next three plots, the histograms show the frequency distribution of within colony variance (==plastic response) while the black vertical lines shows the median variance of this and the red line is the variance as a whole. Arrows are specific formations.

```{r, echo=FALSE}

#Colony.Form$Unique.Colony

#AZ ARE

par(mfrow=c(3,1), mar=c(3,4,2,2))

Sd.Area.Zooid=tapply(log(Measurements$Zooid.area),Measurements$Unique.Colony, var, na.rm=TRUE) #variance of each colony

Sd.Area.Zooid=as.data.frame(Sd.Area.Zooid)

Sd.Area.Zooid$Unique.Colony=rownames(Sd.Area.Zooid)

VAR=Sd.Area.Zooid

VAR=na.omit(VAR)

VAR=join(VAR, Colony.Form, by="Unique.Colony", type="full")

hist(VAR$Sd.Area.Zooid, col="grey", breaks=20, main="Autozooid area", xlab=" ", ylab="")

med=median(VAR$Sd.Area.Zooid, na.rm=T)

v=var(log(Area.Zooid$Area.Zooid), na.rm=T)

abline(v=v, lwd=2, col="red")

abline(v=med, lwd=2, col="black")

Form.sd=tapply(log(Measurements$Zooid.area),Measurements$Formation_name, var, na.rm=TRUE) #variance of each colony

for (i in 1:6){

arrows(Form.sd[i], 40,Form.sd[i], 35, length=0.05, code=2)}

###

###

#Ovicell AREA

Sd.Area.Ovicell=tapply(log(Measurements$Ovicell.area),Measurements$Unique.Colony, var, na.rm=TRUE) #variance of each colony

Sd.Area.Ovicell=as.data.frame(Sd.Area.Ovicell)

Sd.Area.Ovicell$Unique.Colony=rownames(Sd.Area.Ovicell)

VAR=Sd.Area.Ovicell

VAR=join(Sd.Area.Ovicell,Colony.Form, by="Unique.Colony", type="right")

VAR=na.omit(VAR)

hist(VAR$Sd.Area.Ovicell, col="grey", breaks=20, main="Ovicell area", xlab=" ", ylab="")

med=median(VAR$Sd.Area.Ovicell)

v=var(log(Area.Ovicell.c$Area.Ovicell.c), na.rm=T)

abline(v=v, lwd=2, col="red")

abline(v=med, lwd=2, col="black")

Form.sd=tapply(log(Measurements$Zooid.shape),Measurements$Formation_name, var, na.rm=TRUE) #variance of each colony

for (i in 1:6){

arrows(Form.sd[i], 40,Form.sd[i], 35, length=0.05, code=2)}

#Autozooid Shape

Sd.Shape.Zooid=tapply(log(Measurements$Zooid.shape),Measurements$Unique.Colony, var, na.rm=TRUE) #variance of each colony

Sd.Shape.Zooid=as.data.frame(Sd.Shape.Zooid)

Sd.Shape.Zooid$Unique.Colony=rownames(Sd.Shape.Zooid)

VAR=Sd.Shape.Zooid

VAR=join(Sd.Shape.Zooid,Colony.Form, by="Unique.Colony", type="right")

VAR=na.omit(VAR)

hist(VAR$Sd.Shape.Zooid, col="grey", breaks=20, main="Autozooid shape",

xlab=" ", ylab=" ")

med=median(VAR$Sd.Shape.Zooid)

v=var(log(Shape.Zooid$Shape.Zooid), na.rm=T)

abline(v=v, lwd=2, col="red")

abline(v=med, lwd=2, col="black")

Form.sd=tapply(log(Measurements$Zooid.shape),Measurements$Formation_name, var, na.rm=TRUE) #variance of each colony

for (i in 1:6){

arrows(Form.sd[i], 40,Form.sd[i], 35, length=0.05, code=2)}

```

**Formation by formation**

```{r, echo=FALSE}

#Variance by time Area

Form.sd.a=NULL

Sd.Area.Zooid=tapply(log(Measurements$Zooid.area),Measurements$Unique.Colony, var, na.rm=TRUE) #variance of each colony

Sd.Area.Zooid=as.data.frame(Sd.Area.Zooid)

Sd.Area.Zooid$Unique.Colony=rownames(Sd.Area.Zooid)

VAR=Sd.Area.Zooid

VAR=join(VAR,Colony.Form, by="Unique.Colony", type="right")

VAR=na.omit(VAR)

par(mfrow=c(3,2), mar=c(2,4,2,4))

for (i in 1:6){

temp=VAR[which(VAR$Colony.Form==Formation[i]),]

hist(temp$Sd.Area.Zooid, col="grey", breaks=20, main=paste(time.labels[i]), xlab="", xlim=c(0,0.12), ylim=c(0,12))

med=median(temp$Sd.Area.Zooid, na.rm=T)

abline(v=med, lwd=2, col="black")#median of all the individual variances

temp1=Measurements[which(Measurements$Formation_name==Formation[i]),]

Form.sd.a[i]=var(log(temp1$Zooid.area),na.rm=TRUE) #variance for the whole formation

abline(v=Form.sd.a[i], lwd=2, col="blue")

#list.var=temp$Sd.Area.Zooid[which(!is.na(temp$Sd.Area.Zooid))]

#t.test(list.var, mu=Form.sd, alternative="less")

}

dur=c(0.21, 0.06,0.02, 0.06, 0.03, 0.03)

plot(Form.sd.a, dur)

####

#Variance by time Ovicell area

Form.sd.o=NULL

Sd.Area.Ovicell=tapply(log(Measurements$Ovicell.area),Measurements$Unique.Colony, var, na.rm=TRUE) #variance of each colony

Sd.Area.Ovicell=as.data.frame(Sd.Area.Ovicell)

Sd.Area.Ovicell$Unique.Colony=rownames(Sd.Area.Ovicell)

VAR=Sd.Area.Ovicell

VAR=join(Sd.Area.Ovicell,Colony.Form, by="Unique.Colony", type="right")

VAR=na.omit(VAR)

par(mfrow=c(3,2))

for (i in 1:6){

temp=VAR[which(VAR$Colony.Form==Formation[i]),]

hist(temp$Sd.Area.Ovicell, col="grey", breaks=20, main=paste(time.labels[i]), xlim=c(0, 0.06), xlab="")

med=median(temp$Sd.Area.Ovicell, na.rm=T)

abline(v=med, lwd=2, col="black")#median of all the individual variances

temp1=Measurements[which(Measurements$Formation_name==Formation[i]),]

Form.sd.o[i]=var(log(temp1$Ovicell.area),na.rm=TRUE) #variance for the whole formation

abline(v=Form.sd.o[i], lwd=2, col="blue")

#list.var=temp$Sd.Area.Zooid[which(!is.na(temp$Sd.Area.Zooid))]

#t.test(list.var, mu=Form.sd, alternative="less")

}

####

#Variance by time shape az

Form.sd.s=NULL

Sd.Shape.Zooid=tapply(log(Measurements$Zooid.shape),Measurements$Unique.Colony, var, na.rm=TRUE) #variance of each colony

Sd.Shape.Zooid=as.data.frame(Sd.Shape.Zooid)

Sd.Shape.Zooid$Unique.Colony=rownames(Sd.Shape.Zooid)

VAR=Sd.Shape.Zooid

VAR=join(VAR,Colony.Form, by="Unique.Colony", type="right")

VAR=na.omit(VAR)

par(mfrow=c(3,2))

for (i in 1:6){

temp=VAR[which(VAR$Colony.Form==Formation[i]),]

hist(temp$Sd.Shape.Zooid, col="grey", breaks=20, main=paste(time.labels[i]), xlab="", xlim=c(0, 0.08), ylim=c(0,15))

med=median(temp$Sd.Shape.Zooid, na.rm=T)

abline(v=med, lwd=2, col="black")#median of all the individual variances

temp1=Measurements[which(Measurements$Formation_name==Formation[i]),]

Form.sd.s[i]=var(log(temp1$Zooid.shape),na.rm=TRUE) #variance for the whole formation

abline(v=Form.sd.s[i], lwd=2, col="blue")

#list.var=temp$Sd.Area.Zooid[which(!is.na(temp$Sd.Area.Zooid))]

#t.test(list.var, mu=Form.sd, alternative="less")

}

par(mfrow=c(1,3), mar=c(5,4,4,2), oma=c(3,3,2,1))

dur=c(0.21, 0.06,0.02, 0.06, 0.03, 0.03)

plot(Form.sd.a, dur, pch=20, cex=2, ylab="",xlab="", main="Autozooid Area", cex.main=1.5, cex.axis=1.8)

points( Form.sd.a[1], dur[1], cex=3 , col="red")

cor.test(Form.sd.a, dur)

cor.test(Form.sd.a, dur, method="spearman")

plot(Form.sd.o, dur, pch=20, cex=2,ylab="", xlab="", main="Ovicell Area", cex.main=1.5, cex.axis=1.5)

points( Form.sd.o[1], dur[1], cex=3 , col="red")

cor.test(Form.sd.o, dur, pch=20, cex.axis=1.8)

cor.test(Form.sd.o, dur, method="spearman")

plot(Form.sd.s, dur, pch=20, cex=2,ylab="",xlab="", main="Autozooid Shape", cex.main=1.5, cex.axis=1.8)

points( Form.sd.s[1], dur[1], cex=3 , col="red")

cor.test(Form.sd.s, dur, pch=20)

cor.test(Form.sd.s, dur, method="spearman")

mtext(side=1, "average standard deviation", outer=T, cex=2)

mtext(side =2, "Duration", outer=T, cex=2, padj=0)
